# Omic horizon expression: a database of gene expression based on RNA sequencing data

**DOI:** 10.1186/s12864-023-09781-9

**Published:** 2023-11-08

**Authors:** Yuzhe Hu, Dong Xie, Xixi Li, Wenling Han, Yingyu Chen, Huiying Qi, Pingzhang Wang

**Affiliations:** 1 Department of Immunology, NHC Key Laboratory of Medical Immunology (Peking University), School of Basic Medical Sciences, Peking University Health Science Center, No. 38 Xueyuan Road, Beijing, 100191 China; 2 Peking University Center for Human Disease Genomics, No. 38 Xueyuan Road, Beijing, 100191 China; 3 School of Basic Medical Sciences, Peking University Health Science Center, No. 38 Xueyuan Road, Beijing, 100191 China; 4 Department of Health informatics and Management, School of Health Humanities, Peking University Health Science Center, No. 38 Xueyuan Road, Beijing, 100191 China

**Keywords:** Gene expression, Database, Omic Horizon expression, Rat, Lilrb1, Lilrb3

## Abstract

**Background:**

Gene expression profiles have important significance for gene expression characteristics and further functional studies. More attention has been given to the expression databases in humans and mice, but less attention has been given to rats, while rat models also play an irreplaceable role in biomedical experiments.

**Results:**

To depict the rat gene expression profiles in mRNA expression levels, we analyzed over 2,700 RNA sequencing (RNA-Seq) samples from 48 tissues, 40 primary cell types and 25 cell lines; and then mapped them to the latest version of the rat genome reference, mRatBN7.2. Based on these datasets and reanalysis, we constructed a new database, the Omic Horizon Expression Database (http://immudb.bjmu.edu.cn/expression.html), which allows expressional profile query of over 25,000 rat genes based on non-redundant gene symbols. The database supports requests using gene symbols (or alias), Ensemble and Entrez gene IDs. Gene expression profiles can be queried in three categories: tissues, primary cells and cell lines. Application examples including expression profiling and comparison, as well as identification of novel rat genes, were illustrated to show the utility of the database.

**Conclusions:**

As an omic resource, the Omic Horizon Expression Database provides horizons of gene expression profiles across various tissues and cells, which greatly facilitates the identification of rat genes as well as functional clues.

**Supplementary Information:**

The online version contains supplementary material available at 10.1186/s12864-023-09781-9.

## Background

Gene expression profiles play an important role in biological research. The depiction of the gene expression profile helps focus on the role and significance of genes in different diseases and promote the development and application of drugs. Multiple high-throughput sequencing (HTS) techniques have been used to obtain gene expression profiles, and the most commonly used methods are DNA microarray and RNA-Seq. Microarrays have been used as a transcriptomics platform since 1990s [1], while the high-throughput RNA-Seq method has emerged only in the past two decades [2]. Compared to microarrays, RNA-Seq has a higher dynamic range for the detection of low-abundance transcripts and does not necessarily rely on a reference genome, allowing for novel transcript and variant detection [3]. The emergence of HTS has accumulated a large amount of data and made it possible to depict gene expression maps more conveniently. Therefore, benefitting from public data, many databases have been constructed for public users to analyze gene expression profiles.

The exploration of gene expression profiles of experimental animals, such as mice and rats, is useful to clarify the conserved nature of genes in different species, which is an essential premise for the safe and effective application of biological experiments in humans. Mice have a wide variety of strains and mature genome modification techniques, making them the most commonly used laboratory animals. However, in some models, rats are better suited for experimental studies than mice. In some respects, such as cognition and behavior, rats are closer to humans than mice. Compared with mice, rats are larger in size and easier to study physiological characteristics, which are suitable for scientific research on behavior, cells, physiology, biochemistry, pharmacology and toxicology [4]. They are important model animals to increase our understanding of common human diseases and are widely used in physiological studies, such as hypertension, diabetes, breast cancer and neurological diseases.

There are many expression profile databases for humans and mice, such as The Human Protein Atlas (HPA, https://www.proteinatlas.org), Genotype-Tissue Expression (GTEx, https://gtexportal.org), RNA Seq Atlas [5], Mouse Gene Expression Database (MGD, https://www.informatics.jax), Mouse Phenome Database (MPD, https://phenome.jax.org), and Tabula Muris [6]. However, the database for querying rat gene expression has not yet been amply reported. The rat BodyMap database performs RNA-Seq from 11 organs of both sexes of different age rats [7]. The Rat Genome Database [8] (RGD, http://rgd.mcw.edu, 1999) was created by the American Academy of Medical Sciences, collecting data on rat genomics, genetics, physiology and more, but without expression profiles. Additionally, there are some comprehensive databases that can satisfy queries of multiple species, such as ArrayExpress [9] and Gene Expression Omnibus (GEO) [10]. However, both databases are mainly used to store omics data. Microarray datasets in GEO can be analyzed directly online by GEO2R [10]; however, no tools aim for HTS datasets in both databases. Therefore, users without informatics skills cannot systematically analyze and compare gene expression profiles, particularly HTS data.

The analysis of RNA-Seq data generally relies on a target genome for reads mapping and a gene transfer format (GTF) file for expressional quantification. The latest genome versions, such as the human genome reference T2T-CHM13 [11], mouse genome reference GRCm39 [12], and rat genome reference mRatBN7.2 [13], comprise more comprehensive gene information, which facilitates the discovery of some previously unnoticed genes. In addition, the gene-level quantification approach generally uses a GTF file containing gene models, with each model representing the structure of transcripts produced by a given gene. Nevertheless, the gene expression profiles for rat databases mentioned above are annotated using a more previous version of the rat genome reference and GTF file. This makes a lack of genes in the results when the previous or incomplete GTF files are used for gene quantification.

Since the latest rat genome reference mRatBN7.2 was published, there has not been a rat gene expression database satisfying the retrieval requirement. In addition, the current rat gene expression databases are focused on tissues and lack expression information on primary cells and cell lines. To address this need, we constructed the Omic Horizon Expression Database, which facilitates gene expression profiling and further functional clues at multiple levels.

## Construction and content

### Data collection

All HTS dataset information about rat samples was derived from GEO DataSets [10] searching (https://www.ncbi.nlm.nih.gov/geo/) according to processes similar to those described previously [14, 15]. The downloaded samples were due to March 2022. RNA-Seq data, with RNA being total RNA or polyA RNA, from Rattus norvegicus were retained. The tissue and cell sources were manually annotated based on sample characteristics and sorted in ascending order. Then, the top several samples of each type of tissue and cell were selected. The selected sample size accounts for approximately 10% of the total sample. Because the dataset information was associated with the Sequence Read Archive (SRA), the SRA run (SRR) IDs were extracted from the selected dataset information. Based on the accession numbers, RNA-Seq data were downloaded via SRAtools from the SRA database (https://www.ncbi.nlm.nih.gov/sra/) and used for subsequent analysis.

### Data processing

The sra format files were converted to FASTQ format files by the fastq-dump command. The program fastp [16] (https://github.com/OpenGene/fastp) was used for quality control of raw reads according to the standard pipeline. Read alignment to the reference genome of rat mRatBN7.2 was performed by the STAR program [17]. The GTF file (v105) was downloaded from the ENSEMBLES website (http://ftp.ensembl.org/pub/release-105/gtf/rattus_norvegicus/). The rat reference sequences (https://ftp.ncbi.nlm.nih.gov/refseq/R_norvegicus/) was used to help gene symbol annotation. Raw counts of each gene were first calculated with featureCounts [18], and the transcripts per kilobase million (TPM) values (see below) were further calculated and used for the subsequent analysis. During the process, samples with fewer than 5,000 detected genes were removed.

### TPM value calculation

To eliminate the effects of sequencing depth and gene length, we used the TPM value to measure the gene expression level. A TPM value is calculated by the following formula: TPM_i_ = ($\frac{\text{N}\text{i}}{\text{L}\text{i}}$)*10^6^/$\left(\sum _{i=1}^{n}\frac{\text{N}\text{i}}{\text{L}\text{i}}\right)$, where N_i_ indicates the count of reads mapping to gene i and L_i_ indicates the length of exons of gene i.

### Construction of the database

The Omic Horizon Expression database was based on the MVT (Model/View/Template) design pattern and implemented based on the Python web framework-Django. The data were stored and managed by a MySQL relational database (version 8.0.17). Data box plotting via Highchart. Omic Horizon Expression runs on an Apache web server (version 2.4.54). It accesses the database using mysqlclient.

### Molecular cloning of Lilrb1 and Lilrb3

Male Sprague-Dawley rats were obtained from Peking University Health Science Center, Department of Laboratory Animal Science. Rats were sacrificed by carbon dioxide. Tissues were isolated and temporarily stored in TRIzol reagent (Trans#ET111-01). Total RNA in tissues was extracted and reverse transcribed into cDNA. The molecules were cloned using nested polymerase chain reaction (nested-PCR), and the primers are shown in Table 1. The outer primers were labeled F1 and R1 for the forward and reverse primers, respectively, while the inner primers were labeled F2 and R2.

**Table 1:** The primers for molecular cloning

Gene	Primers	
*Lilrb1*	F1:CAGAAAAGCTCTGTAGCTCTG	R1:GAAGAGTGTGGGTTCAGGAA
	F2:CCCAAGCTTCTCTCAAGCAGAGTTGCA	R2:CCGCTCGAGGGAATCACCTCATCATAA
*Lilrb3*	F1:CAGAGCCTCATAATATGACATCC	R1:GCAGGCATATTTCTTATCTCAGAC
	F2:CCCAAGCTTCAGCCTGTGTAGACCATA	R2:CCGCTCGAGTGAAGATCTGAGAATACA
*Gapdh*	F: ATGACTCTACCCACGGCAA	R:GGTTCACACCCATCACAAAC

## Utility and discussion

### Basic description of the database

**Fig. 1:**
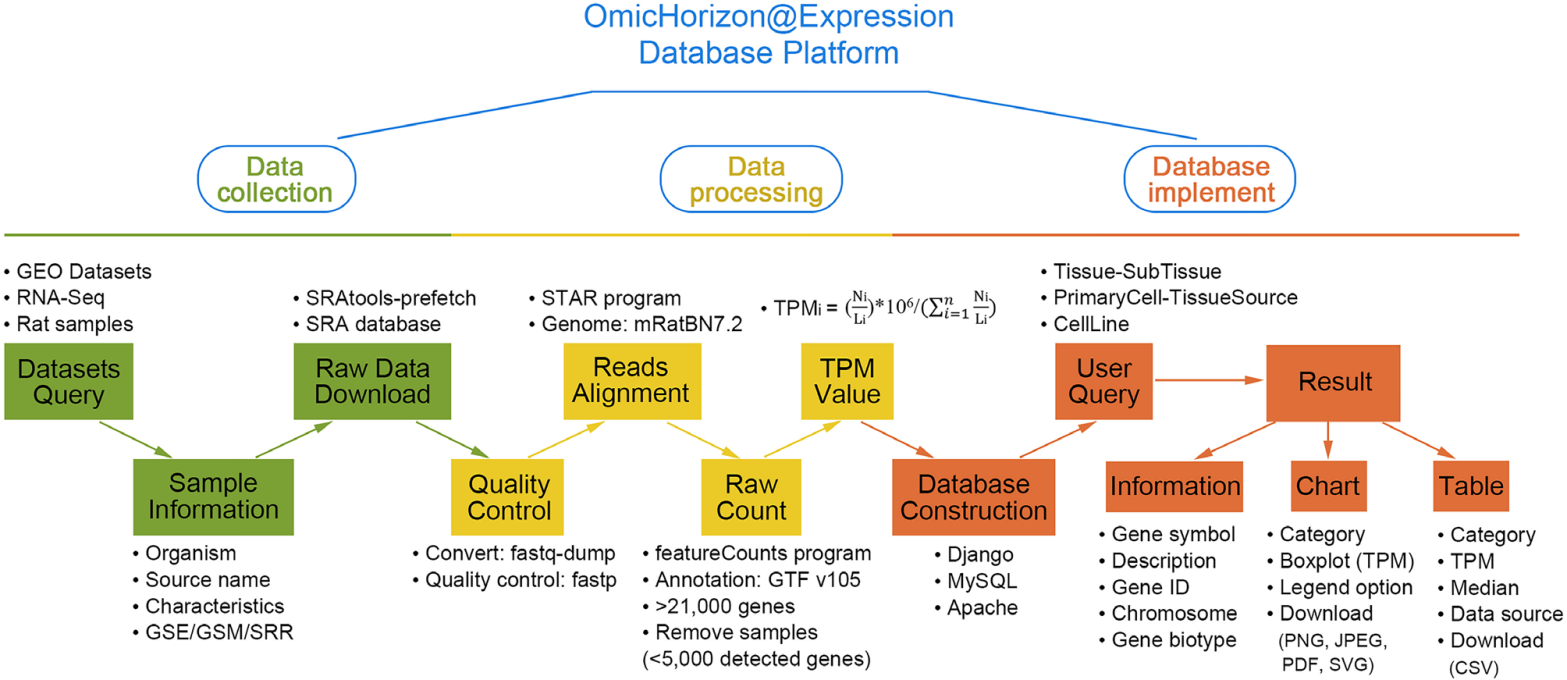
Workflow of the Omic Horizon Expression database development. The Omic Horizon Expression database development includes three major steps: data collection, data processing and database construction. The key points of each step are shown in frames with surrounding information

In this study, we reanalyzed the sequencing data, mapped the reads to the reference genome mRatBN7.2, and then quantified gene expression using the v105 version of the GTF file. A new database for rat gene expression, the Omic Horizon Expression Database (or abbreviated as OmicHorizon@Expression), was constructed (Fig. 1), containing the majority of tissues and cells of publicly available HTS data for rats. To date, it contains 2,762 samples in terms of the sequencing run data (Additional file 1), which are categorized into 48 tissues, 40 primary cell types, and 25 cell lines. In addition, some tissues were further divided into several different subtissues according to the anatomical site, while some primary cells were described in detail based on different tissue sources. The summary of datasets collected in the database is shown in Table 2, and the description of cell lines is shown in Table 3. It is available to query the expression profiles of over 30,000 rat genes based on Ensemble gene IDs by the Omic Horizon Expression database, which supports requests using gene symbols (or alias), Ensemble and Entrez gene IDs.

**Table 2:** The RNA-Seq sample information stored in the Horizon Expression database

	Catalog	Sample size	Catalog	Sample size	Catalog	Sample size
**Tissue**	Achilles tendon	32	Alveolar bone	6	Aorta	8
	Adrenal gland	66	Adipose	23	Bladder	6
	Bone	5	Bone marrow	135	Brain	547
	Cartilage	6	Dorsal root ganglia	12	Breast	18
	Embryo	8	Gingiva	10	Ear	4
	Kidney	218	Patellar tendon	27	Heart	66
	Muscle	139	Large intestine	6	Lung	107
	Prostate	9	Pancreatic islet	8	Colon	5
	Placenta	18	Plantaris tendon	8	Eye	12
	Retina	8	Salivary gland	2	Liver	74
	Skin	29	Small intestine	8	Nerve	12
	Spinal cord	33	Spleen	64	Penis	3
	Stomach	3	Sperm	10	Ovary	5
	Testis	32	Urogenital sinus	4	Uterus	43
	Trachea	4	Urethral tissue	6	Vagina	4
	Thymus	66	Whole blood	6	Yolk sac	8
**Primary** **Cell**	Aortic smooth muscle cell	12	Alveolar type-I-like epithelial cell	5	Astrocyte	18
	Alveolar type 2 cell	3	Breast epithelial cell	6	B-cell	24
	Cardiomyocyte	18	CD45 + immune cell	6	CD4 + T-cell	12
	Cardiac fibroblast	28	Embryonic stem cell	4	Germ cell	8
	Hepatocyte	21	Hepatic stellate cell	4	Leukocyte	2
	Fibroblast-like synoviocyte	12	Oligodendrocyte precursor cell	8	Neural stem cell	8
	Microglia	33	Mesenchymal stem cell	18	Neuron	30
	Macrophage	53	Neural progenitor cell	8	Osteoblast	18
	Oligodendrocyte	12	Retinal ganglion cell	8	Oocyte	8
	Theca-interstitial cell	4	Proximal tubule epithelial cell	5	White blood cell	8
	Pyramidal neuron	16	Renal tube epithelial cell	10	PBMC	37
	Pancreatic beta cell	5	Trophoblast stem cell	8	Sertoli cell	4
	Schwann cell	6	Vascular endothelial cell	18	T-cell	64
	Ventricular myocyte	24				
**Cell** **Line**	AR42J	4	FRT	3	GH4C1	2
	H9C2	24	INS-1	22	INS-1E	5
	IEC-18	6	N27	12	OLN93	4
	Odora cell	15	PAIII	6	PAC1	12
	PC12	27	PCCL3	7	R3327-AT1	10
	RN46A	9	RN46A-B14	6	Rcho1	6
	RPE-J	10	RBL-2H3	2	RASMC	8
	rMC-1	8	SIRMu-1	5	S16	6
	Walker-256	14				

**Table 3:** Description of rat cell lines

Cell Line	Description
AR42J	Pancreatic acinar carcinoma
FRT	Fischer rat thyroid epithelial cell line
GH4C1	Pituitary tumor cell line
H9C2	Embryonic cardiomyoblast cell line
IEC-18	Normal epithelial cells of the rat ileum
INS-1	Insulinoma β cell line
INS-1E	Insulinoma β cell line
N27	Dopaminergic neuronal cell
Odora cell	Olfactory neuron
OLN93	Oligodendrocytes cell line
PAC1	Pulmonary artery smooth muscle cell line
PAIII	Prostate cancer cell
PC12	Neural crest origin pheochromocytoma
PCCL3	Follicular thyroid cell line
R3327-AT1	Prostate cancer cell
RASMC	Rat aortic smooth muscle cell
RBL-2H3	Basophilic leukemia cell line
Rcho1	Choriocarcinoma cell line
rMC-1	Retinal Müller cell line
RN46A	Serotonergic neuronal cell line
RN46A-B14	Serotonergic neuronal cell line (BDNF-overexpression)
RPE-J	Retinal pigment epithelial (RPE) cell line
S16	Primary Schwann cells derived cell line
SIRMu-1	Immortalized rat Müller-1 cell line
Walker-256	Breast carcinoma cell line

### Query of the database

**Fig. 2:**
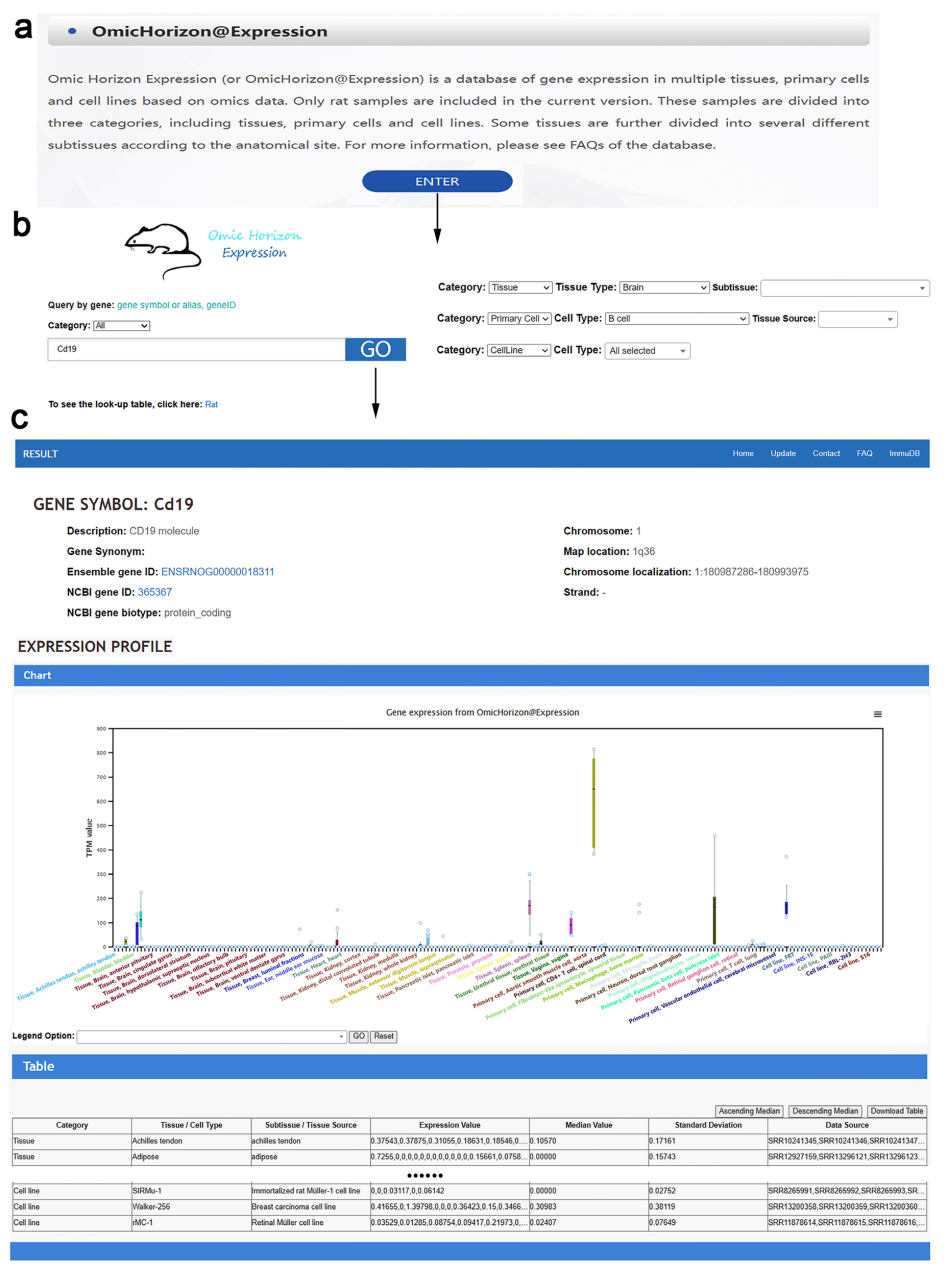
A simplified example of navigating the database. (**a**) The page to enter the OmicHorizon@Expression database. (**b**) The interface of the OmicHorizon@Expression database. (**c**) The result page of the queried gene expression profiles includes three sections: brief information, expression profile indicated by boxplots, and data table that can be downloadable. The data table can be sorted by median. In the second section, the legend option helps users select their interested tissues and cells from the boxplots to refine illustration; the reset button helps return to the initial display result

The interface is simple and easy to understand (Fig. 2a,b). On the homepage, we can select a search type from the drop-down menu as tissue, primary cell and cell line. Then, an interesting tissue or cell type is selected before the new drop-down menu pops up for further selection of subtissue or cell sources. When ‘All’ is selected, all tissues or cells under the layer will be queried to show the result. Then, we entered the query gene symbol or gene ID and clicked on the button ‘GO’ to enter the result page for the gene expression profile. Please note that not all legends on the *x*-axis will be displayed when there are too many retrieved tissues or subtissues, for example, when “All” is selected in the first step. In this situation, the legend option helps users refine illustration when the interested legends are selected from the drop-down list box (Fig. 2c), moreover, the full retrieved results are also shown in a table, which is downloadable.

### Understand the query result

The results page includes three sections (Fig. 2c). The first section is the basic information of the queried gene, such as gene description, gene alias, gene IDs, gene biotype and chromosome location. The second section indicates the gene expression profile shown in box plots. In each plot, the *x*-axis represents the queried tissues, primary cells or cell lines, while the *y*-axis represents the expressional TPM values. A box plot consists of five data nodes, which arrange a set of data from largest to smallest, as the upper edge (Q3 + 1.5*IQR), Q3 (75th percentile), median (50th percentile), Q1 (25th percentile) and lower edge (Q1-1.5*IQR), which are calculated for each profile. The values that overflow the range from the upper edge to the lower edge are identified as the outliers, and are shown as circles in the plot. The upper and lower edges are set to the maximum and minimum values, respectively, when there are no outliers. Each value represents a sample source in the box plots. The third section is a table form showing the gene expressional TPM values of the queried gene. The box plot and table files are downloadable.

### Functions of the database

The Omic Horizon Expression Database provides gene expression profiles in multiple tissues and subtissues that are beneficial for gene functional clues. The “subtissue” catalog in this database provides a more detailed expression profile. For example, the “brain” tissues comprise subtissues from different brain regions, such as the amygdala, anterior pituitary, forebrain, superficial zone and thalamus; the “breast” tissues are divided into subtissues “basal fractions” and “luminal fractions” based on sample location. There are a total of 78 subtissues that are mainly derived from the brain, kidney and muscle tissues. This function offers multiple possibilities for the retrieval and presentation of gene expression.

Expression profiles in primary cells and cell lines help to arrange further functional studies, such as gene overexpression, knockdown or knockout. From the current database version, the primary cells are derived from a total of 33 tissues and subtissues. Some primary cells have multiple sources, such as macrophages from bone marrow, brain, lung and peritoneum. Therefore, differential expression can be analyzed among different tissue sources in the same cell types.

We used several application cases in the next three sections to show how to use the Omic Horizon Expression database to solve biological problems based on gene expression profiles.

### Application case 1: conventional gene expression profiling

**Fig. 3:**
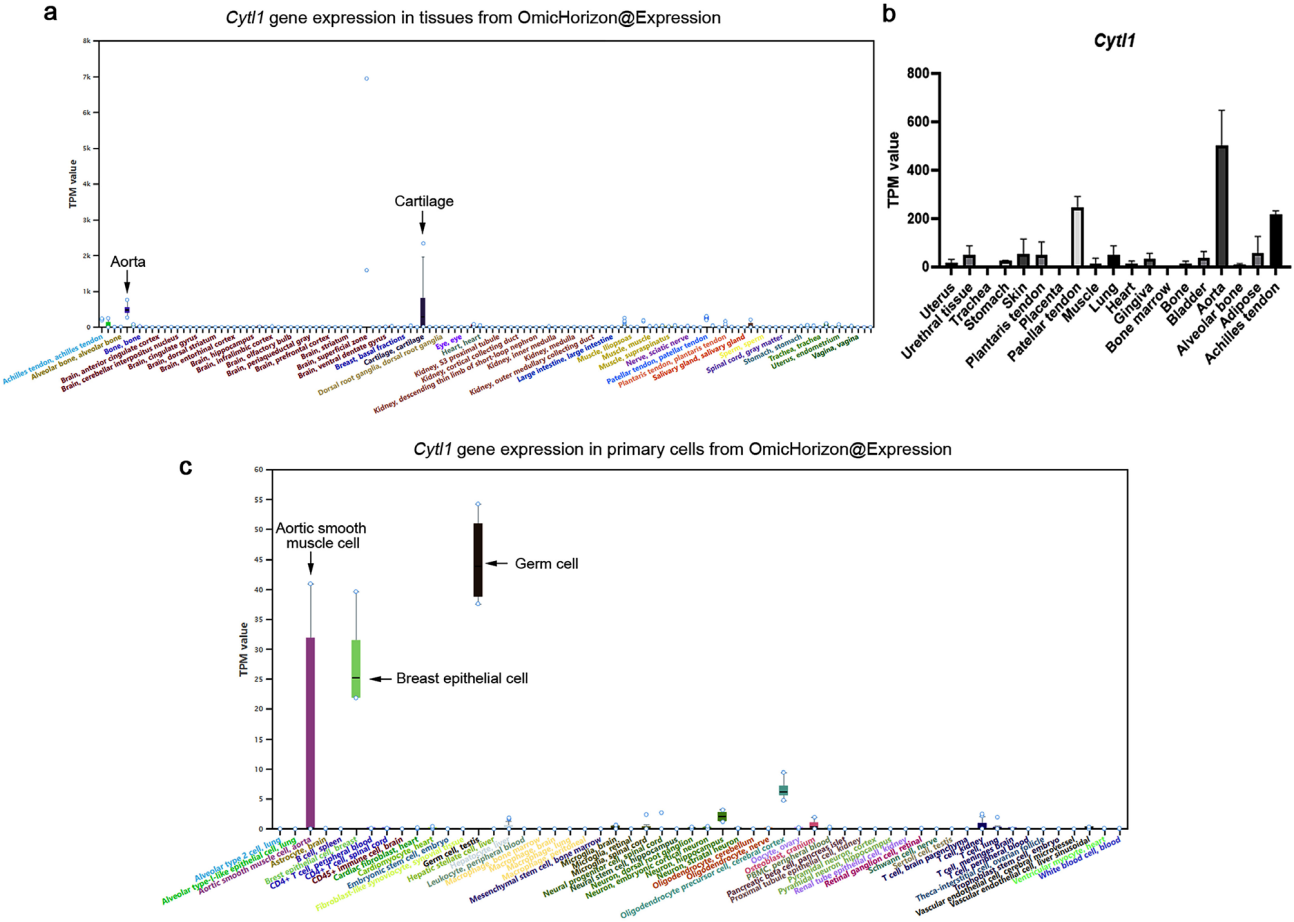
Expression profile of rat Cytl1. (**a**) *Cytl1* expression profiles in rat tissues derived from OmicHorizon@Expression. (**b**) *Cytl1* expression profiles in rat tissues of interest. It is locally drawn according to expression values downloaded from OmicHorizon@Expression. (**c**) *Cytl1* expression profiles in primary cells derived from OmicHorizon@Expression.

This is the general usage to show differential gene expression in various tissues, primary cells and cell lines. For example, cytokine-like protein 1 (CYTL1), also called protein C17, is a secretory protein originally identified in human CD34^+^ cells with a predicted cytokine and interleukin structure characteristics [19]. Sequence alignment showed that CYTL1 genes are conserved in humans, rats and mice [20]. Studies have shown that human *CYTL1* is highly expressed in the aorta, placenta, and trachea [20] and that mouse *Cytl1* is highly expressed in the cartilage, trachea, lung, and heart [21]. Using OmicHorizon@Expression, we found that rat *Cytl1* is highly expressed in cartilage and aorta (Fig. 3a), indicating conservative expression among these species and further suggesting a conservative functional role. The queried expressional values of *Cytl1* can be downloaded, which provides the chance to users to generate their expression profiles of interest (Fig. 3b). In primary cells, *Cytl1* showed relatively high expression in rat germ cells, breast epithelial cells and aortic smooth muscle cells (Fig. 3c).

### Application case 2: species-specific gene expression profiling

**Fig. 4:**
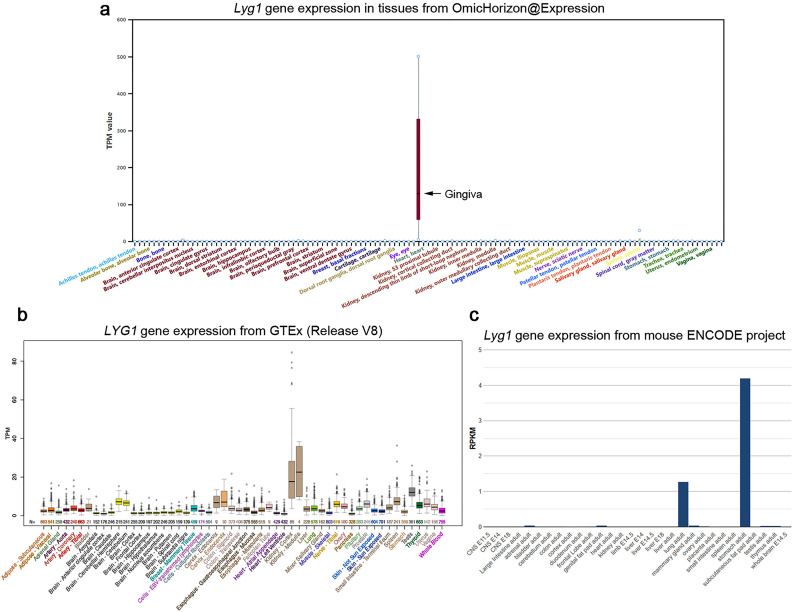
Expression profile of LYG1 genes. (**a**) *Lyg1* expression profiles in rat tissues derived from OmicHorizon@Expression. (**b**) *LYG1* expression profiles in human tissues derived from GTEx. (**c**) *Lyg1* expression profiles in mouse tissues derived from the mouse ENCODE project

Considering the large number of gene expression databases in humans and mice, it is possible to determine whether there is species-specific expression based on the current rat database. For example, lysozyme G-like 1 (LYG1) is a classical secretory protein identified through immunogenomics and belongs to the lysozyme G family [22]. The Omic Horizon Expression database shows that rat *Lyg1* is highly expressed in the gingiva (Fig. 4a). The gene expression profile database indicates that human *LYG1* is highly expressed in the kidney (Fig. 4b) while mouse *Lyg1* is highly expressed in the stomach (Fig. 4c). The change in the expression profiles suggests that *LYG1* gene should evolve actively, which may result in functional discrimination in different species.

### Application case 3: novel genes identification

**Fig. 5:**
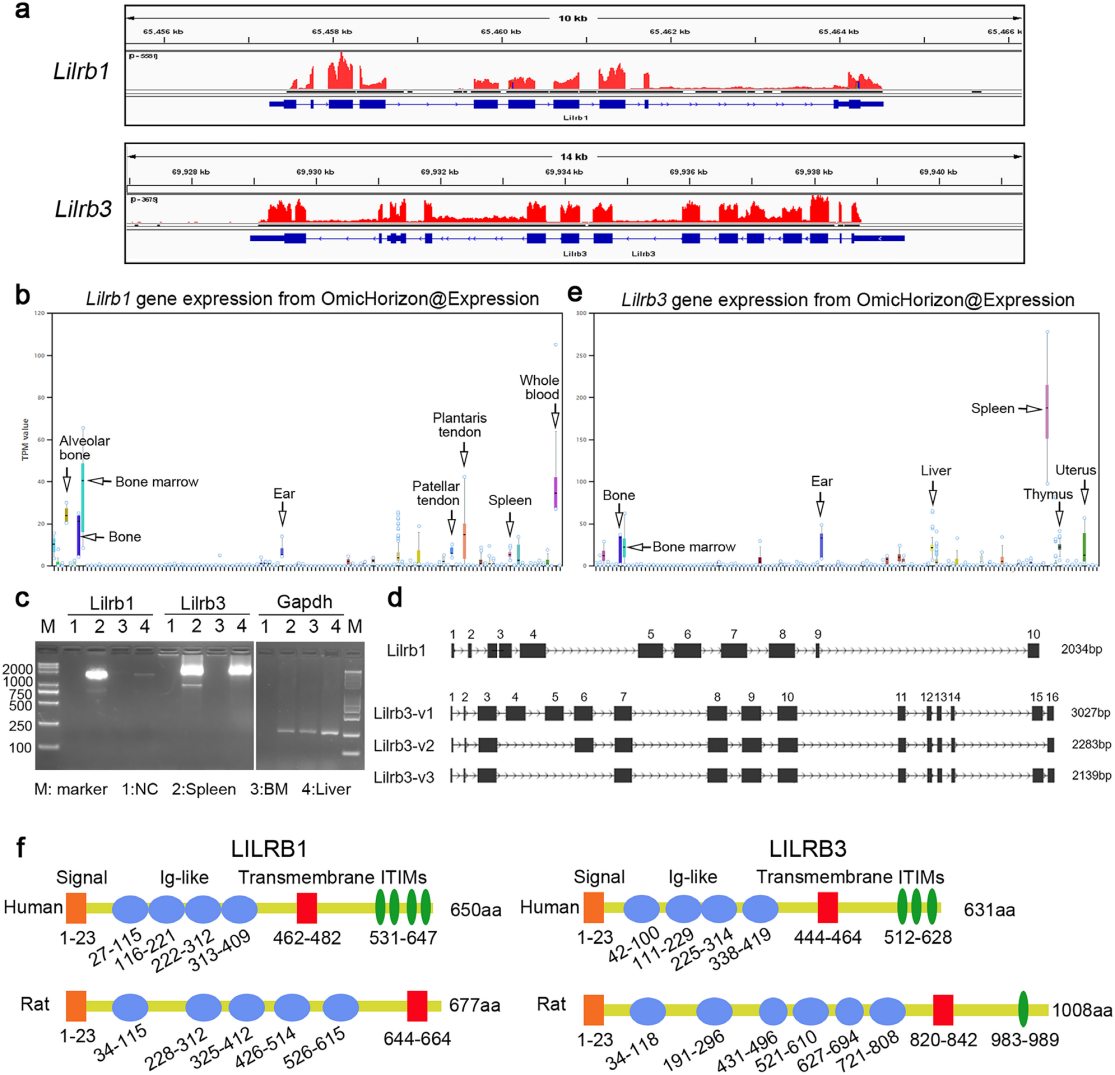
Identification of rat *Lilrb1* and *Lilrb3* genes. (**a**) HTS data support the transcriptional evidence of the rat genes *Lilrb1* and *Lilrb3*. The reference genome mRatBN7.2 was used for read alignment, and the result was viewed by the Integrative Genomics Viewer (IGV) browser. The sequencing data used for *Lilrb1* were merged by 30 samples that were randomly selected from 135 bone marrow samples. The sequencing data used for *Lilrb3* were merged from 35 spleen samples. (**b**) *Lilrb1* expression profiles in rat tissues derived from OmicHorizon@Expression. (**c**) Validation of *Lilrb1* and *Lilrb3* expression in rat tissues by nested-PCR. Tissues are indicated by the numbers as shown. (**d**) The genomic structure of *Lilrb1* and *Lilrb3* based on the amplified sequences. Exon numbers are indicated. The sequencing results were confirmed to be correct and supported by multiple clones. (**e**) *Lilrb3* expression profiles in rat tissues derived from OmicHorizon@Expression. (**f**) Sketch maps of the domains and motifs of LILRB1 and LILRB3 in humans and rats. The human protein motifs are based on the annotation in the UniProt database (https://www.uniprot.org/), while the rat protein motifs are predicted from the SMART web server (http://smart.embl-heidelberg.de/) and protein alignment

The database uses the latest genome and the relevant GTF version, which are useful for identifying novel genes. Immune checkpoint therapy (ICT), which is designed to block inhibitory signals mediated by immune checkpoint molecules, such as PD-1 and CTLA-4, has revolutionized the field of cancer immunotherapy because of its clinical success in many cancers [23]. However, only a minority of patients benefit from current immune checkpoint inhibitors, highlighting the need to identify novel drug targets, including novel promising inhibitory receptors. Leukocyte immunoglobulin-like receptor subfamily B (LILRB) proteins (LILRBs 1–5) contain cytoplasmic immunoreceptor tyrosine-based inhibitory motifs (ITIMs) and transduce a negative signal in multiple cell types in the tumor microenvironment, providing novel opportunities for anti-cancer immunotherapy [24, 25]. Identification of the homologous genes of LILRBs in other species, such as mice and rats, contributes to their functional studies, target validation and drug development. However, the homologous genes of LILRBs in mice and rats have not been fully identified. In the NCBI reference gene and sequence database, there are known rat *Lilrb2* and *Lilrb4* mRNA sequences but only predicted rat *Lilrb1* and *Lilrb3* genes by automated computational annotation deposited in the database. In addition, rat *Lilrb1* and *Lilrb3* genes are not yet recorded by the UCSC Genome Browser (http://genome.ucsc.edu) and are not currently searchable. *Lilrb1* is also lacking in the previous rat GTF versions. Therefore, this prompted us to use HTS data combined with PCR to verify the existence and exon structures of *Lilrb1* and *Lilrb3* in the rat genome (Fig. 5).

HTS data from rat tissues support the transcriptional evidence of the rat genes *Lilrb1* and *Lilrb3* (Fig. 5a). The Omic Horizon expression database revealed that *Lilrb1* is highly expressed in bone marrow, spleen, blood, bone, and tendons (Fig. 5b). We successfully cloned *Lilrb1* from the spleen (Fig. 5c). There was one transcript of *Lilrb1* containing complete open reading frames (ORFs) (Fig. 5d & Additional file 2). However, the sequence similarity between the *Lilrb1* cloned transcript and the predicted transcript in the NCBI GenBank database is 91% in nucleotides (Additional file 3) and 85% in amino acids. The sequence inconsistencies may be due to the differences in animal strains, as Brown Norway rat in the NCBI reference genome but Sprague-Dawley rat in our experiment were used, suggesting that there should be active evolution of *Lilrb1*.

Similarly, the Omic Horizon Expression database shows that *Lilrb3* is highly expressed in the spleen, liver, thymus, bone and ear (Fig. 5e). We successfully cloned *Lilrb3* from the spleen and liver and confirmed the existence of rat *Lilrb3* (Fig. 5c). *Lilrb3* has three transcript variants, including *Lilrb3*-v1, -v2 and -v3, with different exon combinations (Fig. 5d & Additional file 4). The similarities between these cloned transcripts and predicted transcripts of *Lilrb3* in the NCBI GenBank database are all 100% at either the nucleic acid or protein level.

Interestingly, sequence alignments based on rat *Lilrb1* and *Lilrb3* mRNA sequences further confirm the loss of both genes in mice because of multiple pre-stop codons in the deduced coding regions in the homologous genomic region. The protein similarities between human and rat LILRB1 and LILRB3 are 32.5% and 40.6%, respectively (Additional file 5), with similar domains and motifs (Fig. 5f). Therefore, the successful identification of rat *Lilrb1* and *Lilrb3* will contribute to functional studies based on rat models.

## Conclusions

In this study, we constructed a new gene expression database named the Omic Horizon Expression database. It supports gene expression profiling and comparison in 48 tissues, 40 primary cell types and 25 cell lines via the query of gene symbols (or alias), Ensemble and Entrez gene ID for a total of 30,560 rat genes. Through the database, we verified the inexistence of *Lilrb1* and *Lilrb3* in the mouse genome but their existence in the rat genome, which provided the basis for functional studies and drug development in rat models. In the current version, only rat data can be queried, however, multiple species will be considered in the future, especially for primary cells and cell line data, to which other existing databases often receive less attention. In addition, considering the new gene annotation in the more frequently updated GTF files rather than the reference genome version, as well as the accumulation of public data leading to other tissue and cell types that have not yet been covered in the current study, we will regularly update the database.

### Electronic supplementary material

Below is the link to the electronic supplementary material.

**Additional file 1**: Brief information of 2,762 samples based on the sequencing run data. Rat strain, age, sex and the associated PubMed IDs are indicated when the information is available on the NCBI web pages

**Additional file 2**: Nucleotide and deduced amino acid sequence of rat Lilrb1. The ORF of rat *Lilrb1* is underlined and the deduced amino acid sequence is shown below. The boxed letters represent the sequences of the restriction endonucleases *HindIII* and *XhoI*. The nucleotide sequence of *Lilrb1* has been submitted to GenBank with the accession number OP709921

**Additional file 3**: Nucleotide sequence alignment of cloned and predicted rat Lilrb1. The nucleotide sequence alignment of cloned and predicted (XM_003748711) rat Lilrb1 was generated using NCBI Blast (https://blast.ncbi.nlm.nih.gov/)

**Additional file 4**: Nucleotides and deduced amino acid sequences of rat Lilrb3. The ORFs of *Lilrb3*-v1/v2/v3 are underlined, and the deduced amino acid sequences are shown below. The boxed letters represent the sequences of the restriction endonucleases *HindIII* and *XhoI*. The accession numbers of *Lilrb3* submitted to GenBank are OP709922 (*Lilrb3*-v1), OP709923 (*Lilrb3*-v2) and OP709924 (*Lilrb3*-v3)

**Additional file 5**: Protein sequence alignment of human and rat LILRB1 and LILRB3. The sequence alignment of human and rat LILRB1 and LILRB3 was generated using the Clustal Omega program (https://www.ebi.ac.uk/Tools/msa/clustalo/). The symbols below the sequence alignment are explained as follows: An * (asterisk) indicates positions that have a single, fully conserved residue. A : (colon) indicates conservation of strongly similar properties. A. (period) indicates the conservation of weakly similar properties. A - (dash) represents a gap in the alignment

Supplementary Material 6

## Data Availability

Omic Horizon Expression Database is free at http://immudb.bjmu.edu.cn/expression.html.

## List of abbreviations

HTS: High-throughput sequencing
GEO: Gene Expression Omnibus
GTF: Gene transfer format
SRA: Sequence Read Archive
TPM: Transcripts per kilobase million
PCR: Polymerase chain reaction
CYTL1: Cytokine-like protein 1
LYG1: Lysozyme G-like 1
LILRBs: Leukocyte immunoglobulin-like receptor subfamily B
ITIMs: Immunoreceptor tyrosine-based inhibitory motifs
ORFs: Open reading frames
