# Supplementary material for: Omic horizon expression: a database of gene expression based on RNA sequencing data
Source: BMC Genomics. 2023 Nov 8;24:674. doi: 10.1186/s12864-023-09781-9 (PMC10634139; doi:10.1186/s12864-023-09781-9)
Supplement: Supplementary file 2 — Additional file 2: Nucleotide and deduced amino acid sequence of rat Lilrb1. The ORF of rat Lilrb1 is underlined and the deduced amino acid sequence is shown below. The boxed letters represent the sequences of the restriction endonucleases HindIII and XhoI. The nucleotide sequence of Lilrb1 has been submitted to GenBank with the accession number OP709921 [file 12864_2023_9781_MOESM2_ESM.pdf]

Lilrb1

```
1         aagcttctctcaagcagagttgcagtctcggagaggagatgccATGACATTCACCTTCACAGCCCTGCTCTGTCTTGGACTGACTCTGGGCCTCTGGATCCCAGTGCTGACAGGGTCC
   1                                                 M  T  F  T  F  T  A  L  L  C  L  G  L  T  L  G  L  W  I  P  V  L  T  G  S
76        CTCCCTAAACCTATCCTCAGAGCACAGCCAGATTCTGTGGTCTCAATGGGAACTAAGGTGACCTTCATTTGTGAGGAGACCATTGGAGCCAAAGAGTCTTATCTCTATAGAAATGGACAC
   26     L  P  K  P  I  L  R  A  Q  P  D  S  V  V  S  M  G  T  K  V  T  F  I  C  E  E  T  I  G  A  K  E  S  Y  L  Y  R  N  G  H
196       CGAGTTTCAAAGAACCATCAGAAGCCAACAAACAAGACTGAATTCTCATTCTCAAATGTAGGCCAGCAAAGTGCAGGGCAATATCACTGTTCCTACAGGACTCAGACTAAATCATCAGAC
   66     R  V  S  K  N  H  Q  K  P  T  N  K  T  E  F  S  F  S  N  V  G  Q  Q  S  A  G  Q  Y  H  C  S  Y  R  T  Q  T  K  S  S  D
316       TACAGTGAGCCCCTGGAGCTAGTGGTCACAGGAGCCTACTCGAAACCCAGCCTTTCAGCTCAGACCAACCCTGTGGGGACCTCAGGAGGGTATGTCACCCTCAAATGTGAGTCCCAGCAT
   106    Y  S  E  P  L  E  L  V  V  T  G  A  Y  S  K  P  S  L  S  A  Q  T  N  P  V  G  T  S  G  G  Y  V  T  L  K  C  E  S  Q  H
436       TTCGATCACACATTGATTCTTACTGTAGAAGGACCACAGAAGCTCTCCTGGAGGAAGAACCCAGAGTGTCCTGACTATACTTGGATGTGCCGGGCCCTGTTATCTGTGGGCCCTTTGACC
   146    F  D  H  T  L  I  L  T  V  E  G  P  Q  K  L  S  W  R  K  N  P  E  C  P  D  Y  T  W  M  C  R  A  L  L  S  V  G  P  L  T
556       TCCAACCAGAGATGGATAGTCAGATGCTACAGCTATGAAAGAAACAGACCACAACTGTGGTCAGCTCCTAGTGAACCAGTGGAGATCCTAGTGTCAGGGAAACTCCAAAAACCAACCATC
   186    S  N  Q  R  W  I  V  R  C  Y  S  Y  E  R  N  R  P  Q  L  W  S  A  P  S  E  P  V  E  I  L  V  S  G  K  L  Q  K  P  T  I
676       AAGGCTGAACCAGGCTCTGTGATCCGCTCTGGAAAGGCCATAACCATATGGTGTCAGGGGGACCTTGATGCAGAAATATATTTTCTGCATAAGGAGGGAAGCCACAATACACAGAGCACA
   226    K  A  E  P  G  S  V  I  R  S  G  K  A  I  T  I  W  C  Q  G  D  L  D  A  E  I  Y  F  L  H  K  E  G  S  H  N  T  Q  S  T
796       CAGACCCTACAACAGCCTGGGAACAAGGCCAAGTTCTTCATCTCTTCTGTGACACAAGGCCATGCAGGGCAGTATCGCTGTTACTGTTACAGCTCAGCTGGATGGTCAGAGCCCAGTGAC
   266    Q  T  L  Q  Q  P  G  N  K  A  K  F  F  I  S  S  V  T  Q  G  H  A  G  Q  Y  R  C  Y  C  Y  S  S  A  G  W  S  E  P  S  D
916       ACCCTGGAGCTGGTGGTGACAGGAATCTACAACTACTATCCACTCAGGCTGTCAGGACTGCCCAGGCCAGTGGTGCCAGAGGGAGGGAACGTGACACTCCACTGTACCTCACACAGCAAC
   306    T  L  E  L  V  V  T  G  I  Y  N  Y  Y  P  L  R  L  S  G  L  P  R  P  V  V  P  E  G  G  N  V  T  L  H  C  T  S  H  S  N
1036      TATGATAAATTCATTCTCACCAAGGAAGATCAGAAGTTCACCAGCTCACTAGACGCAGAGTATATATCTTCTACTAGTCAATACCAAGCAACGTTTGTTATAGGACCCATGACCCCAAAC
   346    Y  D  K  F  I  L  T  K  E  D  Q  K  F  T  S  S  L  D  A  E  Y  I  S  S  T  S  Q  Y  Q  A  T  F  V  I  G  P  M  T  P  N
1156      TACTCAGGGACATTCAGATGTTATGGTTACAACAAGCATACCCCACAGTTGTGGTCAGTACCCAGTGAGCTCCTAAAAATACTCATCTCAGGACCATCAAGGAAGCCCTCTCTGCTAAGT
   386    Y  S  G  T  F  R  C  Y  G  Y  N  K  H  T  P  Q  L  W  S  V  P  S  E  L  L  K  I  L  I  S  G  P  S  R  K  P  S  L  L  S
1276      CATCAAGGCCATATACTTGATCCTGGAATGAACCTCACACTGCAGTGTTACTCTGACACCAACTATGACAAATTTGCTCTATATAAAGAGGGGGGAACTGACATCATACAAACCTCTAGC
   426    H  Q  G  H  I  L  D  P  G  M  N  L  T  L  Q  C  Y  S  D  T  N  Y  D  K  F  A  L  Y  K  E  G  G  T  D  I  I  Q  T  S  S
1396      CAGTGGACCAAGGCTGGCCTCTGCATGGCCAACTTCACACTGGGCTATGTGAGACAATTCACTGGAGGCCAATACAGATGCTATGGTTCACACAACCTCTCCTCTTTGTTGTCGGCCTCC
   466    Q  W  T  K  A  G  L  C  M  A  N  F  T  L  G  Y  V  R  Q  F  T  G  G  Q  Y  R  C  Y  G  S  H  N  L  S  S  L  L  S  A  S
1516      AGTGACCCCCTGGACATCCTGATCACAGGACAGCTTCATCACACTCCTTCCCTCTCAGTGATGCCCAACTCCACAGTCCACTCAGGAGAGAATGTGACCCTGATGTGTTGGTCAACGTAC
   506    S  D  P  L  D  I  L  I  T  G  Q  L  H  H  T  P  S  L  S  V  M  P  N  S  T  V  H  S  G  E  N  V  T  L  M  C  W  S  T  Y
1636      TCTGTGGACACTTTCATTCTGTCTAAGGAGGGATCAAGCCAGCCACCCCTTCGAATAAGATCCAAGTTCCAAAATCAACAGAATCAATCAGAATTCTCCATAAATGCTGTGACCTCCACC
   546    S  V  D  T  F  I  L  S  K  E  G  S  S  Q  P  P  L  R  I  R  S  K  F  Q  N  Q  Q  N  Q  S  E  F  S  I  N  A  V  T  S  T
1756      CACTCAGGCACCTACAAGTGCTATGGTTCTCAAGATTCATCTCTCTACCTTCTGTCATTTTCCAGTGCCCCTGTGGAGCTCAGAGTCTCAGGACCCATTGAAGCCTCCAGCTGGCCAACT
   586    H  S  G  T  Y  K  C  Y  G  S  Q  D  S  S  L  Y  L  L  S  F  S  S  A  P  V  E  L  R  V  S  G  P  I  E  A  S  S  W  P  T
1876      AAAAGGTACATCACAACAGCCGCTCCAGAGAACCCGGATCACACAATGGAGAATCTCATCAGGATGGGGATGGCCATCCTGGTCCTCATAGTCCTTTCAATTCTAGCTGCTGAGGCCTGG
   626    K  R  Y  I  T  T  A  A  P  E  N  P  D  H  T  M  E  N  L  I  R  M  G  M  A  I  L  V  L  I  V  L  S  I  L  A  A  E  A  W
1996      CAAAGCCATAGACAGTCCCACCATACAGCTGGGAAATAAtctaaagagaaagaagtacatctttcaatggtttacactctgggaaatgaatttgatgatccaaaattttctgtcagaaaa
   666    Q  S  H  R  Q  S  H  H  T  A  G  K  *
          aatgtattgtgatagaatgtccagggagcaaaattgtttggggttctgaggaaagaagttttatgatgaggtgattccctcgag
```
