# Supplementary material for: Omic horizon expression: a database of gene expression based on RNA sequencing data
Source: BMC Genomics. 2023 Nov 8;24:674. doi: 10.1186/s12864-023-09781-9 (PMC10634139; doi:10.1186/s12864-023-09781-9)
Supplement: Supplementary file 3 — Additional file 3: Nucleotide sequence alignment of cloned and predicted rat Lilrb1. The nucleotide sequence alignment of cloned and predicted (XM_003748711) rat Lilrb1 was generated using NCBI Blast (https://blast.ncbi.nlm.nih.gov/) [file 12864_2023_9781_MOESM3_ESM.pdf]

Lilrb1

```
cloned_seq     ATGACATTCACCTTCACAGCCCTGCTCTGTCTTGGACTGACTCTGGGCCTCTGGATCCCAGTGCTGACAGGGTCCCTCCCTAAACCTATCCTCAGAGCACAGCCAGATTCTGTGGTCTCA  120
               ||||| ||||||||||||||||||||||||||||||||||||||||||||||||||||||||||||||||||||||||||| ||||||||||||| ||||||||||||||||||||||
predicted_seq  ATGACCTTCACCTTCACAGCCCTGCTCTGTCTTGGACTGACTCTGGGCCTCTGGATCCCAGTGCTGACAGGGTCCCTCCCTAAGCCTATCCTCAGAGTACAGCCAGATTCTGTGGTCTCA  120

cloned_seq     ATGGGAACTAAGGTGACCTTCATTTGTGAGGAGACCATTGGAGCCAAAGAGTCTTATCTCTATAGAAATGGACACC----GAGT----TT-CAAAGAACCATCAGAAGCCAACAAACAAG  231
               |||||||||| ||||||||||||||||||||||||||||||||||||||||||| ||||||||||||||||| |||    |||     || |||||||| ||||||||||||||||||||
predicted_seq  ATGGGAACTACGGTGACCTTCATTTGTGAGGAGACCATTGGAGCCAAAGAGTCTGATCTCTATAGAAATGGAAACCTACAGAGAAGAGTTCCAAAGAACAATCAGAAGCCAACAAACAAG  240

cloned_seq     ACTGAATTCTCATTCTCAAATGTAGGCCAGCAAAGTGCAGGGCAATATCACTGTTCCTACAGGACTCAGACTAAATCATCAGACTACAGTGAGCCCCTGGAGCTAGTGGTCACAGGAGCC  351
               |||||||||| ||| | |||||||||||| |||| |||||||||||||||||||||||||| | |||||  ||||||||||||||||||||||||||||||||||||||||||||||||
predicted_seq  ACTGAATTCTTATTTTTAAATGTAGGCCATCAAAATGCAGGGCAATATCACTGTTCCTACAAGTCTCAGGGTAAATCATCAGACTACAGTGAGCCCCTGGAGCTAGTGGTCACAGGAGCC  360

cloned_seq     TACTCGAAACCCAGCCTTTCAGCTCAGACCAACCCTGTGGGGACCTCAGGAGGGTATGTCACCCTCAAATGTGAGTCC-CAGCATTTCGATCACACATTGATTCTTACTGTAGAAGGACC  470
               ||||||||||||||||||||||  ||| |||| ||||||| ||||||||||||||||||||||||||||||||||| || || ||||  |||||||||||||||||||||||||||||| |
predicted_seq  TACTCGAAACCCAGCCTTTCAGTCCAGTCCAATCCTGTGGTGACCTCAGGAGGGTATGTCACCCTCAAATGTGAGCCCTCA-CATTATGATCACACATTGATTCTTACTGTAGAAGGATC  479

cloned_seq     ACAGAAGCTCTCCTGGAGGAAGAACCCAGAGTGTCCTGACTATACTTGGATGTGCCGGGCCCTGTTATCTGTGGGCCCTTTGACCTCCAACCAGAGATGGATAGTCAGATGCTACAGCTA  590
                ||||| | ||||||||| || |  || |||||    |||| ||| ||| ||| |  ||||||||  ||||||||||||||||||||||||||||| |||||| ||||||||||||||||
predicted_seq  TCAGAAACCCTCCTGGAGGCAGGAAACACAGTGTAACCACTACACTGGGAAGTGTCATGCCCTGTTCCCTGTGGGCCCTTTGACCTCCAACCAGAGGTGGATATTCAGATGCTACAGCTA  599

cloned_seq     TGAAAGAAACAGACCACAACTGTGGTCAGCTCCTAGTGAACCAGTGGAGATCCTAGTGTCAGGGAAACTCCAAAAACCAACCATCAAGGCTGAACCAGGCTCTGTGATCCGCTCTGGAAA  710
               |||||  |||| ||||||| |||||||||||||||||||||||||||||| |||| |||||||||||||| |||||||||||||||||||||||||||||||||||||| | ||||||||
predicted_seq  TGAAACTAACACACCACAAGTGTGGTCAGCTCCTAGTGAACCAGTGGAGCTCCTGGTGTCAGGGAAACTGCAAAAACCAACCATCAAGGCTGAACCAGGCTCTGTGATGCACTCTGGAAG  719

cloned_seq     GGCCATAACCATATGGTGTCAGGGGGACCTTGATGCAGAAATATATTTTCTGCATAAGGAGGGAAGCCACAATACACAGAGCACACAGACCCTACAACAGCCTGGGAACAAGGCCAAGTT  830
               |||||| ||||| |||||||||||||||||| ||||||||||||||||||||| |||||||||||||||||||||||||||||||||||||||||||||||||||||||||||||||||||
predicted_seq  GGCCATGACCATCTGGTGTCAGGGGGACCTGGATGCAGAAATATATTTTCTGTATAAGGAGGGAAGCCACAATACACAGAGCACACAGACCCTACAACAGCCTGGGAACAAGGCCAAGTT  839

cloned_seq     CTTCATCTCTTCTGTGACACAAGGCCATGCAGGGCAGTATCGCTGTTACTGTTACAGCTCAGCTGGATGGTCAGAGCCCAGTGACACCCTGGAGCTGGTGGTGACAGGAATCTACAACTA  950
               ||||||| |||||||||||||| ||||||||||||| ||| ||||||||||||||||||||||||| |||||||||||||||||||||||||||||||||||||||||||||| ||||||||
predicted_seq  CTTCATCCCTTCTGTGACACAAAGCCATGCAGGGCAATATGGCTGTTACTGTTACAGCTCAGCTGGTTGGTCAGAGCCCAGTGACACCCTGGAGCTGGTGGTGACAGGAATGTACAACTA  959

cloned_seq     CTATCCACTCAGGCTGTCAGGACTGCCCAGGCCAGTGGTGCCAGAGGGAGGGAACGTGACACTCCACTGTACCTCACACAGCAACTATGATAAATTCATTCTCACCAAGGAAGATCAGAA  1070
               ||||||||||| |||||||||||||||||| || ||||||||||||||||||||||||||||||||||||||||||| | | ||||||||||||||||||||||||||||||||||||||
predicted_seq  CTATCCACTCATGCTGTCAGGACTGCCCAGCCCTGTGGTGCCAGAGGGAGGGAACGTGACACTCCACTGTACCTCACTCCGAAACTATGATAAATTCATTCTCACCAAGGAAGATCAGAA  1079

cloned_seq     GTTCACCAGCTCACTAGACGCAGAGTATATATCTTCTACTAGTCAATACCAAGCAACGTTTGTTATAGGACCCATGACCCCAAACTACTCAGGGACATTCAGATGTTATGGTTACAACAA  1190
               |||||||| |||||||||| ||||||||||| |||||||| | ||| |||||||   ||||||  | |||||||||||||||||||||||||||||||||||||||||||||||| ||||
predicted_seq  GTTCACCAACTCACTAGACACAGAGTATATACCTTCTACTGGACAACACCAAGCCCTGTTTGTATTGGGACCCATGACCCCAAACTACTCAGGGACATTCAGATGTTATGGTTACTACAA  1199

cloned_seq     GCATACCCCACAGTTGTGGTCAGTACCCAGTGAGCTCCTAAAAATACTCATCTCAGGACCATCAAGGAAGCCCTCTCTGCTAAGTCATCAAGGCCATATACTTGATCCTGGAATGAACCT  1310
               | ||||||||||||||||||||||||||||||||||||||||||||||||||||||||||||  |||||||| |||||||||||||||||||| | ||||||  |||||||||||| |||
predicted_seq  GAATACCCCACAGTTGTGGTCAGTACCCAGTGAGCTCCTAAAAATACTCATCTCAGGACCATTGAGGAAGCCATCTCTGCTAAGTCATCAAGGACGTATACTAAATCCTGGAATGAGCCT  1319

cloned_seq     CACACTGCAGTGTTACTCTGACACCAACTATGACAAATTTGCTCTATATAAAGAGGGGGGAACTGACATCATACAAACCTCTAGCCAGTGGACCAAGGCTGGCCTCTGCATGGCCAACTT  1430
               ||||||||||||||||||||||| ||| ||||||||||||||| || || ||||| |||||| |||||||||||||  ||||||||||||||||||||||||||||| ||  ||||||||
predicted_seq  CACACTGCAGTGTTACTCTGACATCAATTATGACAAATTTGCACTGTACAAAGAAGGGGGAGCTGACATCATACAGTCCTCTAGCCAGTGGACCAAGGCTGGCCTCTCCAAAGCCAACTT  1439

cloned_seq     CACACTGGGCTATGTGAGACAATTCACTGGAGGCCAATACAGATGCTATGGTTCACACAACCTCTCCTCTTTGTTGTCGGCCTCCAGTGACCCCCTGGACATCCTGATCACAGGACAGCT  1550
               ||||||||| ||||| |  || | |||||||||||||||||||||||||||| |||||||||||||||||  ||||||||||||||||||||||||||||||||||||||||||||| |
predicted_seq  CACACTGGGATATGTAAACCACTCCACTGGAGGCCAATACAGATGCTATGGTGCACACAACCTCTCCTCTGAGTTGTCGGCCTCCAGTGACCCCCTGGACATCCTGATCACAGGACAGAT  1559

cloned_seq     TCATCACACTCCTTCCCTCTCAGTGATGCCCAACTCCACAGTCCACTCAGGAGAGAATGTGACCCTGATGTGTTGGTCAACGTACTCTGTGGACACTTTCATTCTGTCTAAGGAGGGATC  1670
               | || |||| |||||||||||||||||||||||||||||||| |||||||||||||||||||||||||||||||||||||| |||||||||||||||||||||||||| |||||||||||
predicted_seq  TTATGACACACCTTCCCTCTCAGTGATGCCCAACTCCACAGTACACTCAGGAGAGAATGTGACCCTGATGTGTTGGTCAAAGTACTCTGTGGACACTTTCATTCTGTCAAAGGAGGGATC  1679

cloned_seq     AAGCCAGCCACCCCTTCGAATAAGATCCAAGTTCCAAAATCAACAGAATCAATCAGAATTCTCCATAAATGCTGTGACCTCCACCCACTCAGGCACCTACAAGTGCTATGGTTCTCAAGA  1790
               | ||||||||||||||||||  ||  || |||||||| |||||||| | || |||||||||||||||| |  |||||||| ||||  | ||| ||||||||||| ||||||||||||||  ||
predicted_seq  AGGCCAGCCACCCCTTCGACGAAAGTCAAAGTTCCAAGATCAACAGTACCAGTCAGAATTCTCCATGAGAGCTGTGACATCCAAGCTCTCGGGCACCTACAGGTGCTATGGTTCTCCCGA  1799

cloned_seq     TTCATCTCTCTACCTTCTGTCATTTTCCAGTGCCCCTGTGGAGCTCAGAGTCTCAGGACCCATTGAAGCCTCCAGCTGGCCAACTAAAAGGTACATCACAACAGCCGCTCCAGAGAACCC  1910
                ||||||||||||||  |||||||| ||||||||||||||||||||| |||||||| | ||| |||||||||||||||||| ||||||||| |||||||||||| |||||||| |||| 
predicted_seq  CTCATCTCTCTACCTGTTGTCATTTGCCAGTGCCCCTGTGGAGCTCATAGTCTCAGAATCCATCGAAGCCTCCAGCTGGCCAGCTAAAAGGTTCATCACAACAGCCACTCCAGAAAACCA  1919

cloned_seq     GGATCACACAATGGAGAATCTCATCAGGATGGGGATGGCCATCCTGGTCCTCATAGTCCTTTCAATTCTAGCTGCTGAGGCCTGGCAAAGCCATAGACAGTCCCACCATACAGCTGGGAA  2030
               |||||||||||||||||||||||||||||||||||||||  ||||||||||||||||||||||| ||||||||||||| |||||||||||| ||||||||| |||||||  ||||||| ||
predicted_seq  GGATCACACAATGGAGAATCTCATCAGGATGGGGATGGCTGTCCTGGTCCTCATAGTCCTTTCGATTCTAGCTGCTGTGGCCTGGCAAAGTCATAGACAGACCCACCACGCAGCTGGCAA  2039

cloned_seq     ATAA  2034
               ||||
predicted_seq  ATAA  2043
```
