# Supplementary material for: Omic horizon expression: a database of gene expression based on RNA sequencing data
Source: BMC Genomics. 2023 Nov 8;24:674. doi: 10.1186/s12864-023-09781-9 (PMC10634139; doi:10.1186/s12864-023-09781-9)
Supplement: Supplementary file 4 — Additional file 4: Nucleotides and deduced amino acid sequences of rat Lilrb3. The ORFs of Lilrb3-v1/v2/v3 are underlined, and the deduced amino acid sequences are shown below. The boxed letters represent the sequences of the restriction endonucleases HindIII and XhoI. The accession numbers of Lilrb3 submitted to GenBank are OP709922 (Lilrb3-v1), OP709923 (Lilrb3-v2) and OP709924 (Lilrb3-v3) [file 12864_2023_9781_MOESM4_ESM.pdf]

Lilrb3-v1

```
1     aagcttcagcctgtgtagaccatacactttgtgtctgtacatttggcaagaagacaagctctgaaaacctgcagcaactggaagatgccATGGTTTCTACTCTCACAGCCCTTGCTTAT
   1                                                                                          M  V  S  T  L  T  A  L  A  Y
31    CTTGGATTGATCCTGGGCTCCGAGAACCCAGTCCTGTCAGGTGCCTTCACTAAACCTACAATAAAGATGGTGCCAAGCAATGTGGTAACTACTGGGAAACAGGTGACCATCTTCTGTGAA
   11 L  G  L  I  L  G  S  E  N  P  V  L  S  G  A  F  T  K  P  T  I  K  M  V  P  S  N  V  V  T  T  G  K  Q  V  T  I  F  C  E
151   GGGTCCTCACATGCCAAAGAATACCGTCTCCACAAAGAAGGAAGTCCCGATTACCTGACACCAACAACCTTTCTGGAAACTGAGAACAAGGCCAAGTTCTCAATCTCACCAATTCAATGG
   51 G  S  S  H  A  K  E  Y  R  L  H  K  E  G  S  P  D  Y  L  T  P  T  T  F  L  E  T  E  N  K  A  K  F  S  I  S  P  I  Q  W
271   AATAATGCAGGACAATACTGGTGTTCGTATAGAAGCCTGACTAACAAATTACGACAAAGTGACATCATGGAGCTGGTGGTGACAGGAGTTATCCTTGGTGAAGTCACACTGTCTGCCCTT
   91 N  N  A  G  Q  Y  W  C  S  Y  R  S  L  T  N  K  L  R  Q  S  D  I  M  E  L  V  V  T  G  V  I  L  G  E  V  T  L  S  A  L
391   CCCAGCTATGTTGTGACTTCAGGGAGAAATGTGACTCTTCAGTGTGCATCACAAGTAGTATATGATAGGTTCATTCTAATGAAGGAAGATGAGAAGTTCTCCACTGCAATGCCCTCATGG
  131 P  S  Y  V  V  T  S  G  R  N  V  T  L  Q  C  A  S  Q  V  V  Y  D  R  F  I  L  M  K  E  D  E  K  F  S  T  A  M  P  S  W
511   AAAATATACCCTGTACTATGGGGAGCCATTTTCACATTAGGTCCTGTGACCTCAAACCAGAGATGGAGGTTCACATGCTATGGATATTACTTGAGTAGCTCTCTGATATGGTCAAAGCCA
  171 K  I  Y  P  V  L  W  G  A  I  F  T  L  G  P  V  T  S  N  Q  R  W  R  F  T  C  Y  G  Y  Y  L  S  S  S  L  I  W  S  K  P
631   AGTAACCATTTAGAGCTATTGGTCTCGGGGACCCTTCACAAACCCATACTCTGGGCTCATCCAGGTTCCATGGTAACATCAGGGAGCCCTGTGACCATCTGGTGTGAGGGGGACCAAGAA
  211 S  N  H  L  E  L  L  V  S  G  T  L  H  K  P  I  L  W  A  H  P  G  S  M  V  T  S  G  S  P  V  T  I  W  C  E  G  D  Q  E
751   ACACAAACATATGTGCTATATAGACAGGGAAGCCTAGAGTCCTGGAACAGACAGACCCAAAAGGACTATAACAATAAAGCAGAATTCACCATCCCATCTGTGACATATTTGAATGCAGGG
  251 T  Q  T  Y  V  L  Y  R  Q  G  S  L  E  S  W  N  R  Q  T  Q  K  D  Y  N  N  K  A  E  F  T  I  P  S  V  T  Y  L  N  A  G
871   CATTATTTCTGTTACTCTTACACCTCTGCTGGGTGGTCAGAACACAGTGACACCCTGGAGCTGGTGGTTACAGGAGTCTACAAAAAACCCATCTTTTTGGCCCTGAAAAACCCTATTGTA
  291 H  Y  F  C  Y  S  Y  T  S  A  G  W  S  E  H  S  D  T  L  E  L  V  V  T  G  V  Y  K  K  P  I  F  L  A  L  K  N  P  I  V
991   AACTTAGGAGTGACTGTGACCATCTCATGTACCTCAAATCAGAGTTTTAACTGTTTCCTTTTAATGGATGATGATCAGAAAATCTACCGATTCTTTGACTTACAATACTCATATTCTGGA
  331 N  L  G  V  T  V  T  I  S  C  T  S  N  Q  S  F  N  C  F  L  L  M  D  D  D  Q  K  I  Y  R  F  F  D  L  Q  Y  S  Y  S  G
1111  GAATCTCTAGCCCGATTTCAAGTGGGTCCAATAACCTCTAGACAAAGGTGGAGATTCAGATGCTATGGCTATTATACAGATAATCCTCAGGTGTGGTCAGAAGCCAGCGATCCATTGGAT
  371 E  S  L  A  R  F  Q  V  G  P  I  T  S  R  Q  R  W  R  F  R  C  Y  G  Y  Y  T  D  N  P  Q  V  W  S  E  A  S  D  P  L  D
1231  TTCCTGGTCTCAGGGAACCTCCAGAAACCCACCTTGTGGGCTGAGCCAGGTTCAGTGATTGAATCAGGAAATTCTGTGACCATTTGGTGTGAAGGGACCATGGAAACTCAAATATATTTC
  411 F  L  V  S  G  N  L  Q  K  P  T  L  W  A  E  P  G  S  V  I  E  S  G  N  S  V  T  I  W  C  E  G  T  M  E  T  Q  I  Y  F
1351  CTGTATAAAGAAGGAAGCCCATCATCCTGGCTCAGACAAACTCCAAAGGAGCCTGGTAACAAGGCCATGTTCTTCATTGCATTCATGGAAAAGCATAATGCAGGGCAATATCGCTGTTAC
  451 L  Y  K  E  G  S  P  S  S  W  L  R  Q  T  P  K  E  P  G  N  K  A  M  F  F  I  A  F  M  E  K  H  N  A  G  Q  Y  R  C  Y
1471  TGTTACAATTTTGGTGGGTGGTCACAGCACAGTGACACACTGGAGCTGGTGATGACAGGTGTTCACCATGGCAAACCTACTCTTTCAGCCTTTCCCAGTCCTATGGTGACCTCAGGTGGA
  491 C  Y  N  F  G  G  W  S  Q  H  S  D  T  L  E  L  V  M  T  G  V  H  H  G  K  P  T  L  S  A  F  P  S  P  M  V  T  S  G  G
1591  AATGTGACCCTCTATTGTGCCTCATCAACAATATATGATTGGTACATTGTGACTGGGCAAGATTTGAAGTTCTCCAGATTCCAGAGGGCACAGTTCATACCCACCGAGATGTCCCAGGCT
  531 N  V  T  L  Y  C  A  S  S  T  I  Y  D  W  Y  I  V  T  G  Q  D  L  K  F  S  R  F  Q  R  A  Q  F  I  P  T  E  M  S  Q  A
1711  CTGTTCTCTGAGATCTCTGTGGCATCTAGAAAGAAAGGGCCATTTAGATGTTATGGATTCAATGAAAGTACTCCACATCTGTGGTCAGAGGCCAGTAATCCTCTGGAGATACATGTTTCA
  571 L  F  S  E  I  S  V  A  S  R  K  K  G  P  F  R  C  Y  G  F  N  E  S  T  P  H  L  W  S  E  A  S  N  P  L  E  I  H  V  S
1831  GGGGTTTCAAGGAAGCCCTCTCTGCTGAACCAACAAGGCCCTGTTCTGGCCCCTGGAGAGAACCTGACACTCCGGTGTTCCTCTGAGCTCAGTTATGACAGATTTTCTCTATCCAAAGAG
  611 G  V  S  R  K  P  S  L  L  N  Q  Q  G  P  V  L  A  P  G  E  N  L  T  L  R  C  S  S  E  L  S  Y  D  R  F  S  L  S  K  E
1951  GGTAGAAGTGACCTCCCACAGCTCTCTGTCAGCCAGTCACAGACTGGAGAGTATTATGCCAACTTCTTTTATATTCTGTGGATTTCTCTATTGCTGGCCAATATAGATGCTATGGTGCA
  651 G  R  S  D  L  P  Q  L  S  V  S  Q  S  Q  T  G  E  Y  Y  A  N  F  F  L  Y  S  V  D  F  S  I  A  G  Q  Y  R  C  Y  G  A
2071  TCCAGATTTTCTTCTGAGTGGTCAGCCCCCAGTGTTCCTCAAGACATTCTGGTCACAGGACATCCTCCTATTACACCAAGCCTCTCAGTGAATCCAGGCACCATTGTGTCCTCAGGAGAG
  691 S  R  F  S  S  E  W  S  A  P  S  V  P  Q  D  I  L  V  T  G  H  P  P  I  T  P  S  L  S  V  N  P  G  T  I  V  S  S  G  E
2191  AATGTGACCCTTCTCTGTCAATCATCAATACCAATGGACACTTTCTTTCTGTTCAAGAAAGGTGATGCCTATCCCTACATGCACCAAAGATTAAAGTTCCAAGGTCCACAATGTAAGGCA
  731 N  V  T  L  L  C  Q  S  S  I  P  M  D  T  F  F  L  F  K  K  G  D  A  Y  P  Y  M  H  Q  R  L  K  F  Q  G  P  Q  C  K  A
2311  GAATTCTCCCTGAGTGCTGTGACCCCAAACATTGGGGGTATCTTTACGTGCTTTGGTTCTCAAAGCTCATCTCCTTACCTGTTGTCACACCCCAGTGTTCCTGTGGAGATCAAAGTTTCA
  771 E  F  S  L  S  A  V  T  P  N  I  G  G  I  F  T  C  F  G  S  Q  S  S  S  P  Y  L  L  S  H  P  S  V  P  V  E  I  K  V  S
2431  GGACTGGCAAAGTACCAGAAGTCTTTGATATGGGTCTCCGTAGTCTTCTTCCTACTGTTCTTTGTCCTCACCCTCTTCTTTTTCCTCAGACTCTGGCATCAGAACAAGCACAGGAAAGGA
  811 G  L  A  K  Y  Q  K  S  L  I  W  V  S  V  V  F  F  L  L  F  F  V  L  T  L  F  F  F  L  R  L  W  H  Q  N  K  H  R  K  G
2551  GTACAGACAAAGATTGACTTGCAACATCCAGAAGGAGCTGTAGAGCCAAAGAGCATTGTTGGACACCTGCAGAAGAGTTCCAGACCAGCTCCTGCCATCCAGGAAGAAATCTTGTGTGAG
  851 V  Q  T  K  I  D  L  Q  H  P  E  G  A  V  E  P  K  S  I  V  G  H  L  Q  K  S  S  R  P  A  P  A  I  Q  E  E  I  L  C  E
2671  AAAAGATGGCCTGGGAGGGAGATAACTGAGGTCTCAGCATCTGATGCTACTGTGAAGGTCACAAGGTCTGCAGACAATGTGGAGCTAGGCATACTGAGCCAACATCAGGATGACCCCTCC
  891 K  R  W  P  G  R  E  I  T  E  V  S  A  S  D  A  T  V  K  V  T  R  S  A  D  N  V  E  L  G  I  L  S  Q  H  Q  D  D  P  S
2791  ATACACTTGTATGCCCAGGTGAAACCTGCTAGACTCAGAAGGGCACAGACTACCTCTTCTTCCCTGTTTCCAAAGGAATTACAGCACTCTAAACACAGACAGAAAAACAGAGACCAAGTC
  931 I  H  L  Y  A  Q  V  K  P  A  R  L  R  R  A  Q  T  T  S  S  S  L  F  P  K  E  L  Q  H  S  K  H  R  Q  K  N  R  D  Q  V
2911  ATAGATCAGCAGGCTGATACATCTCAGGATTCTCATGCTGTGATTTATGCCCAGCTGCACATCATGACACCAAGACAGGGCAGGCAAACTTCCCTCTTTCCGGGAAGAATATGCTGAatt
  971 I  D  Q  Q  A  D  T  S  Q  D  S  H  A  V  I  Y  A  Q  L  H  I  M  T  P  R  Q  G  R  Q  T  S  L  F  P  G  R  I  C  *
      gagacccttctctgttgtcaaccagtccagagagatctagaatctacattccgtgggagaggaaggactcagccatgtcagaaaatgagatctgtcacaggcacactgtcattgtgcaaa
      aatatctggtgtctagtgtattctgagatcttcactcgag
```

Lilrb3-v2

```
1       aagcttcagcctgtgtagaccatacactttgtgtctgtacatttggcaagaagacaagctctgaaaacctgcagcaactggaagatgccATGGTTTCTACTCTCACAGCCCTTGCTTAT
    1                                                                                            M  V  S  T  L  T  A  L  A  Y
31      CTTGGATTGATCCTGGGCTCCGAGAACCCAGTCCTGTCAGGTGCCTTCACTAAACCTACAATAAAGATGGTGCCAAGCAATGTGGTAACTACTGGGAAACAGGTGACCATCTTCTGTGAA
    11  L  G  L  I  L  G  S  E  N  P  V  L  S  G  A  F  T  K  P  T  I  K  M  V  P  S  N  V  V  T  T  G  K  Q  V  T  I  F  C  E
151     GGGTCCTCACATGCCAAAGAATACCGTCTCCACAAAGAAGGAAGTCCCGATTACCTGACACCAACAACCTTTCTGGAAACTGAGAACAAGGCCAAGTTCTCAATCTCACCAATTCAATGG
    51  G  S  S  H  A  K  E  Y  R  L  H  K  E  G  S  P  D  Y  L  T  P  T  T  F  L  E  T  E  N  K  A  K  F  S  I  S  P  I  Q  W
271     AATAATGCAGGACAATACTGGTGTTCGTATAGAAGCCTGACTAACAAATTACGACAAAGTGACATCATGGAGCTGGTGGTGACAGGAGTCTACAAAAAACCCATCTTTTTGGCCCTGAAA
    91  N  N  A  G  Q  Y  W  C  S  Y  R  S  L  T  N  K  L  R  Q  S  D  I  M  E  L  V  V  T  G  V  Y  K  K  P  I  F  L  A  L  K
391     AACCCTATTGTAAACTTAGGAGTGACTGTGACCATCTCATGTACCTCAAATCAGAGTTTTAACTGTTTCCTTTTAATGGATGATGATCAGAAAATCTACCGATTCTTTGACTTACAATAC
    131 N  P  I  V  N  L  G  V  T  V  T  I  S  C  T  S  N  Q  S  F  N  C  F  L  L  M  D  D  D  Q  K  I  Y  R  F  F  D  L  Q  Y
511     TCATATTCTGGAGAATCTCTAGCCCGATTTCAAGTGGGTCCAATAACCTCTAGACAAAGGTGGAGATTCAGATGCTATGGCTATTATACAGATAATCCTCAGGTGTGGTCAGAAGCCAGC
    171 S  Y  S  G  E  S  L  A  R  F  Q  V  G  P  I  T  S  R  Q  R  W  R  F  R  C  Y  G  Y  Y  T  D  N  P  Q  V  W  S  E  A  S
631     GATCCATTGGATTTCCTGGTCTCAGGGAACCTCCAGAAACCCACCTTGTGGGCTGAGCCAGGTTCAGTGATTGAATCAGGAAATTCTGTGACCATTTGGTGTGAAGGGACCATGGAAACT
    211 D  P  L  D  F  L  V  S  G  N  L  Q  K  P  T  L  W  A  E  P  G  S  V  I  E  S  G  N  S  V  T  I  W  C  E  G  T  M  E  T
751     CAAATATATTTCCTGTATAAAGAAGGAAGCCCATCATCCTGGCTCAGACAAACTCCAAAGGAGCCTGGTAACAAGGCCATGTTCTTCATTGCATTCATGGAAAAGCATAATGCAGGGCAA
    251 Q  I  Y  F  L  Y  K  E  G  S  P  S  S  W  L  R  Q  T  P  K  E  P  G  N  K  A  M  F  F  I  A  F  M  E  K  H  N  A  G  Q
871     TATCGCTGTTACTGTTACAATTTTGGTGGGTGGTCACAGCACAGTGACACACTGGAGCTGGTGATGACAGGTGTTCACCATGGCAAACCTACTCTTTCAGCCTTTCCCAGTCCTATGGTG
    291 Y  R  C  Y  C  Y  N  F  G  G  W  S  Q  H  S  D  T  L  E  L  V  M  T  G  V  H  H  G  K  P  T  L  S  A  F  P  S  P  M  V
991     ACCTCAGGTGGAAATGTGACCCTCTATTGTGCCTCATCAACAATATATGATTGGTACATTGTGACTGGGCAAGATTTGAAGTTCTCCAGATTCCAGAGGGCACAGTTCATACCCACCGAG
    331 T  S  G  G  N  V  T  L  Y  C  A  S  S  T  I  Y  D  W  Y  I  V  T  G  Q  D  L  K  F  S  R  F  Q  R  A  Q  F  I  P  T  E
1111    ATGTCCCAGGCTCTGTTCTCTGAGATCTCTGTGGCATCTAGAAAGAAAGGGCCATTTAGATGTTATGGATTCAATGAAAGTACTCCACATCTGTGGTCAGAGGCCAGTAATCCTCTGGAG
    371 M  S  Q  A  L  F  S  E  I  S  V  A  S  R  K  K  G  P  F  R  C  Y  G  F  N  E  S  T  P  H  L  W  S  E  A  S  N  P  L  E
1231    ATACATGTTTCAGGGGTTTCAAGGAAGCCCTCTCTGCTGAACCAACAAGGCCCTGTTCTGGCCCCTGGAGAGAACCTGACACTCCGGTGTTCCTCTGAGCTCAGTTATGACAGATTTTCT
    411 I  H  V  S  G  V  S  R  K  P  S  L  L  N  Q  Q  G  P  V  L  A  P  G  E  N  L  T  L  R  C  S  S  E  L  S  Y  D  R  F  S
1351    CTATCCAAAGAGGGTAGAAGTGACCTCCCACAGCTCTCTGTCAGCCAGTCACAGACTGGAGAGTATTATGCCAACTTCTTTTTATATTCTGTGGATTTCTCTATTGCTGGCCAATATAGA
    451 L  S  K  E  G  R  S  D  L  P  Q  L  S  V  S  Q  S  Q  T  G  E  Y  Y  A  N  F  F  L  Y  S  V  D  F  S  I  A  G  Q  Y  R
1471    TGCTATGGTGCATCCAGATTTTCTTCTGAGTGGTCAGCCCCCAGTGTTCCTCAAGACATTCTGGTCACAGGACATCCTCCTATTACACCAAGCCTCTCAGTGAATCCAGGCACCATTGTG
    491 C  Y  G  A  S  R  F  S  S  E  W  S  A  P  S  V  P  Q  D  I  L  V  T  G  H  P  P  I  T  P  S  L  S  V  N  P  G  T  I  V
1591    TCCTCAGGAGAGAATGTGACCCTTCTCTGTCAATCATCAATACCAATGGACACTTTCTTTCTGTTCAAGAAAGGTGATGCCTATCCCTACATGCACCAAAGATTAAAGTTCCAAGGTCCA
    531 S  S  G  E  N  V  T  L  L  C  Q  S  S  I  P  M  D  T  F  F  L  F  K  K  G  D  A  Y  P  Y  M  H  Q  R  L  K  F  Q  G  P
1711    CAATGTAAGGCAGAATTCTCCCTGAGTGCTGTGACCCCAAACATTGGGGGTATCTTTACGTGCTTTGGTTCTCAAAGCTCATCTCCTTACCTGTTGTCACACCCCAGTGTTCCTGTGGAG
    571 Q  C  K  A  E  F  S  L  S  A  V  T  P  N  I  G  G  I  F  T  C  F  G  S  Q  S  S  S  P  Y  L  L  S  H  P  S  V  P  V  E
1831    ATCAAAGTTTCAGGACTGGCAAAGTACCAGAAGTCTTTGATATGGGTCTCCGTAGTCTTCTTCCTACTGTTCTTTGTCCTCACCCTCTTCTTTTTCCTCAGACTCTGGCATCAGAACAAG
    611 I  K  V  S  G  L  A  K  Y  Q  K  S  L  I  W  V  S  V  V  F  F  L  L  F  F  V  L  T  L  F  F  F  L  R  L  W  H  Q  N  K
1951    CACAGGAAAGGAGTACAGACAAAGATTGACTTGCAACATCCAGAAGGAGCTGTAGAGCCAAAGAGCATTGTTGGACACCTGCAGAAGAGTTCCAGACCAGCTCCTGCCATCCAGGAAGAA
    651 H  R  K  G  V  Q  T  K  I  D  L  Q  H  P  E  G  A  V  E  P  K  S  I  V  G  H  L  Q  K  S  S  R  P  A  P  A  I  Q  E  E
2071    ATCTTGTGTGAGAAAAGATGGCCTGGGAGGGAGATAACTGAGGTCTCAGCATCTGATGCTACTGTGAAGGTCACAAGGTCTGCAGACAATGTGGAGCTAGGCATACTGGCTGATACATCT
    691 I  L  C  E  K  R  W  P  G  R  E  I  T  E  V  S  A  S  D  A  T  V  K  V  T  R  S  A  D  N  V  E  L  G  I  L  A  D  T  S
2191    CAGGATTCTCATGCTGTGATTTATGCCCAGCTGCACATCATGACACCAAGACAGGGCAGGCAAACTTCCCTCTTTCCGGGAAGAATATGCTGAattgagacccttctctgttgtcaacca
    731 Q  D  S  H  A  V  I  Y  A  Q  L  H  I  M  T  P  R  Q  G  R  Q  T  S  L  F  P  G  R  I  C  *
        gtccagagagatctagaatctacattcccgtggggagaggaaggactcagccatgtcagaaaatgagatctgtcacaggcacactgtcattgtgcaaaaatatctggtgtctagtgtatt
        ctcagatcttcactcgag
```

Lilrb3-v3

```
1          aagcttcagcctgtgtagaccatacactttgtgtctgtacatttggcaagaagacaagctctgaaaacctgcagcaactggaagatgccATGGTTTCTACTCTCACAGCCCTTGCTTAT
    1                                                                                           M  V  S  T  L  T  A  L  A  Y
31         CTTGGATTGATCCTGGGCTCCGAGAACCCAGTCCTGTCAGGTGCCTTCACTAAACCTACAATAAAGATGGTGCCAAGCAATGTGGTAACTACTGGGAAACAGGTGACCATCTTCTGTGAA
    11     L  G  L  I  L  G  S  E  N  P  V  L  S  G  A  F  T  K  P  T  I  K  M  V  P  S  N  V  V  T  T  G  K  Q  V  T  I  F  C  E
151        GGGTCCTCACATGCCAAAGAATACCGTCTCCACAAAGAAGGAAGTCCCGATTACCTGACACCAACAACCTTTCTGGAAACTGAGAACAAGGCCAAGTTCTCAATCTCACCAATTCAATGG
    51     G  S  S  H  A  K  E  Y  R  L  H  K  E  G  S  P  D  Y  L  T  P  T  T  F  L  E  T  E  N  K  A  K  F  S  I  S  P  I  Q  W
271        AATAATGCAGGACAATACTGGTGTTCGTATAGAAGCCTGACTAACAAATTACGACAAAGTGACATCATGGAGCTGGTGGTGACAGGGAACCTCCAGAAACCCACCTTGTGGGCTGAGCCA
    91     N  N  A  G  Q  Y  W  C  S  Y  R  S  L  T  N  K  L  R  Q  S  D  I  M  E  L  V  V  T  G  N  L  Q  K  P  T  L  W  A  E  P
391        GGTTCAGTGATTGAATCAGGAAATTCTGTGACCATTTGGTGTGAAGGGACCATGGAAACTCAAATATATTTCCTGTATAAAGAAGGAAGCCCATCATCCTGGCTCAGACAAACTCCAAAG
    131    G  S  V  I  E  S  G  N  S  V  T  I  W  C  E  G  T  M  E  T  Q  I  Y  F  L  Y  K  E  G  S  P  S  S  W  L  R  Q  T  P  K
511        GAGCCTGGTAACAAGGCCATGTTCTTCATTGCATTCATGGAAAAGCATAATGCAGGGCAATATCGCTGTTACTGTTACAATTTTGGTGGGTGGTCACAGCACAGTGACACACTGGAGCTG
    171    E  P  G  N  K  A  M  F  F  I  A  F  M  E  K  H  N  A  G  Q  Y  R  C  Y  C  Y  N  F  G  G  W  S  Q  H  S  D  T  L  E  L
631        GTGATGACAGGTGTTCACCATGGCAAACCTACTCTTTCAGCCTTTCCCAGTCCTATGGTGACCTCAGGTGGAAATGTGACCCTCTATTGTGCCTCATCAACAATATATGATTGGTACATT
    211    V  M  T  G  V  H  H  G  K  P  T  L  S  A  F  P  S  P  M  V  T  S  G  G  N  V  T  L  Y  C  A  S  S  T  I  Y  D  W  Y  I
751        GTGACTGGGCAAGATTTGAAGTTCTCCAGATTCCAGAGGGCACAGTTCATACCCACCGAGATGTCCCAGGCTCTGTTCTCTGAGATCTCTGTGGCATCTAGAAAGAAAGGGCCATTTAGA
    251    V  T  G  Q  D  L  K  F  S  R  F  Q  R  A  Q  F  I  P  T  E  M  S  Q  A  L  F  S  E  I  S  V  A  S  R  K  K  G  P  F  R
871        TGTTATGGATTCAATGAAAGTACTCCACATCTGTGGTCAGAGGCCAGTAATCCTCTGGAGATACATGTTTCAGGGGTTTCAAGGAAGCCCTCTCTGCTGAACCAACAAGGCCCTGTTCTG
    291    C  Y  G  F  N  E  S  T  P  H  L  W  S  E  A  S  N  P  L  E  I  H  V  S  G  V  S  R  K  P  S  L  L  N  Q  Q  G  P  V  L
991        GCCCCTGGAGAGAACCTGACACTCCGGTGTTCCTCTGAGCTCAGTTATGACAGATTTTCTCTATCCAAAGAGGGTAGAAGTGACCTCCCACAGCTCTCTGTCAGCCAGTCACAGACTGGA
    331    A  P  G  E  N  L  T  L  R  C  S  S  E  L  S  Y  D  R  F  S  L  S  K  E  G  R  S  D  L  P  Q  L  S  V  S  Q  S  Q  T  G
1111       GAGTATTATGCCAACTTCTTTTTATATTCTGTGGATTTCTCTATTGCTGGCCAATATAGATGCTATGGTGCATCCAGATTTTCTTCTGAGTGGTCAGCCCCCAGTGTTCCTCAAGACATT
    371    E  Y  Y  A  N  F  F  L  Y  S  V  D  F  S  I  A  G  Q  Y  R  C  Y  G  A  S  R  F  S  S  E  W  S  A  P  S  V  P  Q  D  I
1231       CTGGTCACAGGACATCCTCCTATTACACCAAGCCTCTCAGTGAATCCAGGCACCATTGTGTCCTCAGGAGAGAATGTGACCCTTCTCTGTCAATCATCAATACCAATGGACACTTTCTTT
    411    L  V  T  G  H  P  P  I  T  P  S  L  S  V  N  P  G  T  I  V  S  S  G  E  N  V  T  L  L  C  Q  S  S  I  P  M  D  T  F  F
1351       CTGTTCAAGAAAGGTGATGCCTATCCCTACATGCACCAAAGATTAAAGTTCCAAGGTCCACAATGTAAGGCAGAATTCTCCCTGAGTGCTGTGACCCCAAACATTGGGGGTATCTTTACG
    451    L  F  K  K  G  D  A  Y  P  Y  M  H  Q  R  L  K  F  Q  G  P  Q  C  K  A  E  F  S  L  S  A  V  T  P  N  I  G  G  I  F  T
1471       TGCTTTGGTTCTCAAAGCTCATCTCCTTACCTGTTGTCACACCCCAGTGTTCCTGTGGAGATCAAAGTTTCAGGACTGGCAAAGTACCAGAAGTCTTTGATATGGGTCTCCGTAGTCTTC
    491    C  F  G  S  Q  S  S  S  P  Y  L  L  S  H  P  S  V  P  V  E  I  K  V  S  G  L  A  K  Y  Q  K  S  L  I  W  V  S  V  V  F
1591       TTCCTACTGTTCTTTGTCCTCACCCTCTTCTTTTTCCTCAGACTCTGGCATCAGAACAAGCACAGGAAAGGAGTACAGACAAAGATTGACTTGCAACATCCAGAAGGAGCTGTAGAGCCA
    531    F  L  L  F  F  V  L  T  L  F  F  F  L  R  L  W  H  Q  N  K  H  R  K  G  V  Q  T  K  I  D  L  Q  H  P  E  G  A  V  E  P
1711       AAGAGCATTGTTGGACACCTGCAGAAGAGTTCCAGACCAGCTCCTGCCATCCAGGAAGAAATCTTGTGTGAGAAAAGATGGCCTGGGAGGGAGATAACTGAGGTCTCAGCATCTGATGCT
    571    K  S  I  V  G  H  L  Q  K  S  S  R  P  A  P  A  I  Q  E  E  I  L  C  E  K  R  W  P  G  R  E  I  T  E  V  S  A  S  D  A
1831       ACTGTGAAGGTCACAAGGTCTGCAGACAATGTGGAGCTAGGCATACTGAGCCAACATCAGGATGACCCCTCCATACACTTGTATGCCCAGGTGAAACCTGCTAGACTCAGAAGGGCACAG
    611    T  V  K  V  T  R  S  A  D  N  V  E  L  G  I  L  S  Q  H  Q  D  D  P  S  I  H  L  Y  A  Q  V  K  P  A  R  L  R  R  A  Q
1951       ACTACCTCTTCTTCCCTGTTTCCAAAGGAATTACAGCACTCTAAACACAGACAGAAAAACAGAGACCAAGTCATAGATCAGCAGGCTGATACATCTCAGGATTCTCATGCTGTGATTTAT
    651    T  T  S  S  S  L  F  P  K  E  L  Q  H  S  K  H  R  Q  K  N  R  D  Q  V  I  D  Q  Q  A  D  T  S  Q  D  S  H  A  V  I  Y
2071       GCCCAGCTGCACATCATGACACCAAGACAGGGCAGGCAAACTTCCCTCTTTCCGGGAAGAATATGCTGAattgagacccttctctgttgtcaaccagtccagagagatctagaatctaca
    691    A  Q  L  H  I  M  T  P  R  Q  G  R  Q  T  S  L  F  P  G  R  I  C  *
           ttccgtgggagaggaaggactcagccatgtcagaaaatgagatctgtcacaggcacactgtcattgtgcaaaaatatctggtgtctagtgtattctgagatcttcactcgag
```
