# Supplementary material for: Omic horizon expression: a database of gene expression based on RNA sequencing data
Source: BMC Genomics. 2023 Nov 8;24:674. doi: 10.1186/s12864-023-09781-9 (PMC10634139; doi:10.1186/s12864-023-09781-9)
Supplement: Supplementary file 5 — Additional file 5: Protein sequence alignment of human and rat LILRB1 and LILRB3. The sequence alignment of human and rat LILRB1 and LILRB3 was generated using the Clustal Omega program (https://www.ebi.ac.uk/Tools/msa/clustalo/). The symbols below the sequence alignment are explained as follows: An * (asterisk) indicates positions that have a single, fully conserved residue. A : (colon) indicates conservation of strongly similar properties. A. (period) indicates the conservation of weakly similar properties. A - (dash) represents a gap in the alignment [file 12864_2023_9781_MOESM5_ESM.pdf]

LILRB1

```
Human   MTPILTVLICLGLSLGPRTHVQAGHLPKPTLWAEPGSVITQGSPVTLRCQGGQETQEYRLYREKKTAPWITRIPQELVKKGQFPIPSITWEHTGRYRCYY   100
Rat     MTFTFTALLCLGLTLGLWIPVLTGSLPKPILRAQPDSVVSMGTKVTFICEETIGAKESYLYRN---GHRVSKNHQKPTNKTEFSFSNVGQQSAGQYHCSY   97
        **  :*.*:****:**    * :* **** * *:*.**:: *: **: *:    ::*  ***:   .  :::  *: .:* :*.:..:  : :*:*:* *

Human   GSDTAGRSESSDPLELVVTGAYIKPTLSAQPSPVVNSGGNVTLQCDSQVAFDGFILCKEGEDEHPQCLN-SQPHARGSSRAIFSVGPVSPSRRWWYRCYA   199
Rat     RTQTKS-SDYSEPLELVVTGAYSKPSLSAQTNPVGTSGGYVTLKCESQHFDHTLILTVEGPQKLSWRKNPECPDYTWMCRALLSVGPLTSNQRWIVRCYS   196
         ::* . *: *:********** **:****..** .*** ***:*:**    . :**  ** :: .    * . *.    .**::****::..:**  ***:

Human   YDSNSPYEWSLPSDLLELLVLGVSKKPSLSVQPGPIVAPEETLTLQCGSDAGYNRFVLYKDGERDFLQLAGAQPQAGLSQANFTLGPVSRSYGGQYRCYG   299
Rat     YERNRPQLWSAPSEPVEILVSGKLQKPTIKAEPGSVIRSGKAITIWCQGDLDAEIYFLHKEGSHNTQSTQTLQQPG--NKAKFFISSVTQGHAGQYRCYC   294
        *: * *  ** **: :*:** *  :**::..:**.:: . :::*: * .* . : :.*:*:*.::  .    *  .  .:*:* :..*::.:.******

Human   AHNLSSEWSAPSDPLDILIAGQF-YDRVSLSVQPGPTVASGENVTLLCQSQGWMQTFLLTKEG-AADDPWRLRSTYQSQKYQAEFPMGPVTSAHAGTYRC   397
Rat     YS--SAGWSEPSDTLELVVTGIYNYYPLRLSGLPRPVVPEGGNVTLHCTSHSNYDKFILTKEDQKFTSSLDAEYISSTSQYQATFVIGPMTPNYSGTFRC   392
            *: ** ***.*:::::* : *  : **  * *.*..* **** * *:.  :.*:****.     ..   .     .:.:*** * :**:*. ::**:**

Human   YGSQSSKPYLLTHPSDPLELVVSGPSGGPSSPTTG----------PTSTSGPEDQPLTPTGSDPQSGLGRHLGVVIGILVAVILLLLLLLLLFLILRHRR   487
Rat     YGYNKHTPQLWSVPSELLKILISGPSRKPSLLSHQGHILDPGMNLTLQCYSDTNYDKFALYKEGGTDIIQTSSQWTKAGLCMANFTLGYVRQFTGGQYRC   492
        ** :. .* * : **: *::::****  **  :              . . . :     . .:  :.: :  .          :.:  : *  :  *   ::*

Human   QGKHWTSTQRKAD------------FQHPAGAVGPEPTDRGLQWRSSPAADAQEENLYAAVKHTQPEDGVEMDTRSPHDEDPQAVTYAEVKHSRPRREMA   575
Rat     YGSHNLSSLLSASSDPLDILITGQLHHTPSLSVMPNSTVHSGENVTLMCWSTYSVDTFILSKEGSSQPPLRIRSKFQNQQNQSEFSINAVTSTHSGTYKC   592
         *.*  *:  .*.            .: *: :* *:.* :. :  :  . .: . : :    *. ..:  :.: ::  :::: . .:     *. ::.    .

Human   SPPSPLSGEFLDTKDRQAEEDRQMDTEAAASEAPQDVTYAQLHSLTLRREATEPPPSQEGPSPAVPSIYATLAIH-----------   650
Rat     YGSQDSSLYLLSFSSAPVELRVSGPIEASSWPTKRYITTAAPENPDHTMENLIRMG-MAILVLIVLSILAAEAWQSHRQSHHTAGK   677
          ..  *  :*. ..  .*   .    **::  : : :* *  ..     *              * ** *: * :
```

LILRB3

```
Human   MTPALTALLCLGLSLGPRTRVQAGPFPKP-----------------------------------------------------------------------   29
Rat     MVSTLTLALAYLGLILGSENPVLSGAFTKPTIKMVPSNVVTTGKQVTIFCEGSSHAKEYRLHKEGSPDYLTPTTFLETENKAKFSISPIQWNNAGQYWCSY   100
        *..:****  *** **... * :*.*.**

Human   ----------------------------------------------------------------------------------------------------   29
Rat     RSLTNKLRQSDIMELVVTGVILGEVTLSALPSYVVTSGRNVTLQCASQVVYDRFILMKEDEKFSTAMPSWKIYPVLWGAIFTLGPVTSNQRWRFTCYGYY   200

Human   -------------------------TLWAEPGSVISWGSPVTIWCQGSQEAQEYRLHKEGSPEPLDRNNPLEPKNKARFSIPSMTEHHAGRYRCHYYSSA   104
Rat     LSSSLIWSKPSNHLELLVSGTLHKPILWAHPGSMVTSGSPVTIWCEGDQETQTYVLYRQGSLESWNRQTQKDYNNKAEFTIPSVTYLNAGHYFCYSYTSA   300
                                  ***.***::: ********:*.**:* * *:::** *. :*:.  : :***.*:***:*  :**:* *: *:**

Human   GWSEPSDPLEMVMTGAYSKPTLSALPSPVVASGGNMTLRCGSQKGYHHFVLMKEGEHQLPRTLDSQQLHSRGFQALFPVGPVTPSHRWRFTCYYYYTNTP   204
Rat     GWSEHSDTLELVVTGVYKKPIFLALKNPIVNLGVTVTISCTSNQSFNCFLLMDD-DQKIYRFFDLQYSYSGESLARFQVGPITSRQRWRFRCYGYYTDNP   399
        **** **.**:*:**.*.** : ** .*:*  * .:*: * *::: * *::.:: *:**.: :::: * :* *  :*     * * ***:*. :**** ** ***:.*

Human   WVWSHPSDPLEILPSG------------------------------------------------------------------------------------   220
Rat     QVWSEASDPLDFLVSGNLQKPTLWAEPGSVIESGNSVTIWCEGTMETQIYFLYKEGSPSSWLRQTPKEPGNKAMFFIAFMEKHNAGQYRCYCYNFGGWSQ   499
         ***..****::* **

Human   ----------------------------------------------------------------------------------------------------   220
Rat     HSDTLELVMTGVHHGKPTLSAFPSPMVTSGGNVTLYCASSTIYDWYIVTGQDLKFSRFQRAQFIPTEMSQALFSEISVASRKKGPFRCYGFNESTPHLWS   599

Human   ------------VSRKPSLLTLQGPVLAPGQSLTLQCGSDVGYNRFVLYKEGERDFLQRPGQQPQAGLSQANFTLGPVSPSNGGQYRCYGAHNLSSEWSA   308
Rat     EASNPLEIHVSGVSRKPSLLNQQGPVLAPGENLTLRCSSELSYDRFSLSKEGRSDLPQLSVSQSQTGEYYANFFLYSVDFSIAGQYRCYGASRFSSEWSA   699
                    ********. ********:.***:*.*::.*:** * ***. *: *  . .*.*:*    *** * .*. * .********  .:******

Human   PSDPLNILMAGQIYDTVSLSAQPGPTVASGENVTLLCQSWWQFDTFLLTKEGAAHPPLRLRSMYGAHKYQAEFPMSPVTSAHAGTYRCYGSYSSNPHLLS   408
Rat     PSVPQDILVTGHPPITPSLSVNPGTIVSSGENVTLLCQSSIPMDTFFLFKKGDAYPYMHQRLKFQGPQCKAEFSLSAVTPNIGGIFTCFGSQSSSPYLLS   799
        ** * :**::*:   * ***.:**. *:************   :***:* *:* *:* ::  *   :  . : :***.:*.**.  .* : *:** **.*:***

Human   HPSEPLELVVSGHSGGSSLPPTGPPSTPGLGRYLEVLIGVSVAFVLLLFLLLFLLLRRQRHS-KHRTSDQRKTDFQRPAGAAETEPKDRGLLRRSSPAAD   507
Rat     HPSVPVEIKVS------------------GLAKYQKSLIWVSVVFFLLFFVLTLFFFLRLWHQNKHRKGVQTKIDLQHPEGAVEPKSIVGHLQKSSRPAPA   882
        *** *:*: **                  **.:* : ** ***.*.**:*:* :::: *  *. ***.. * * *:*:* **.*.:.     * : * **.

Human   VQEENLYAA----------------VKDTQSEDRVELDSQSPHDEDPQAVTYAPVKHSSPRREMASPPSSLSGEFLDTKDRQVEEDRQMDTEAAASEASQ   591
Rat     IQEEILCEKRWPGREITEVSASDATVKVTRSADNVELGILSQHQDDPSIHLYAQVKPARLRRAQTTSSSLFPKELQHSKHRQKNRDQVIDQQADTSQDSH   982
        :*** *                   ** *:* *.***.  * *::**.   ** ** :**  ::..* :. *:  .:*.** :.*: :* :* :*: *:

Human   DVTYAQLHSLTLRRKATEPPPSQEGEPPAEPSIYATLAIH   631
Rat     AVIYAQLHIMTPRQGRQTSLFPGRIC--------------   1008
         * ***** :* *:     .  .  .
```
