# Supplementary figures and images for: Omic horizon expression: a database of gene expression based on RNA sequencing data
Source: BMC Genomics. 2023 Nov 8;24:674. doi: 10.1186/s12864-023-09781-9 (PMC10634139; doi:10.1186/s12864-023-09781-9)

# Full images for Figure 5c

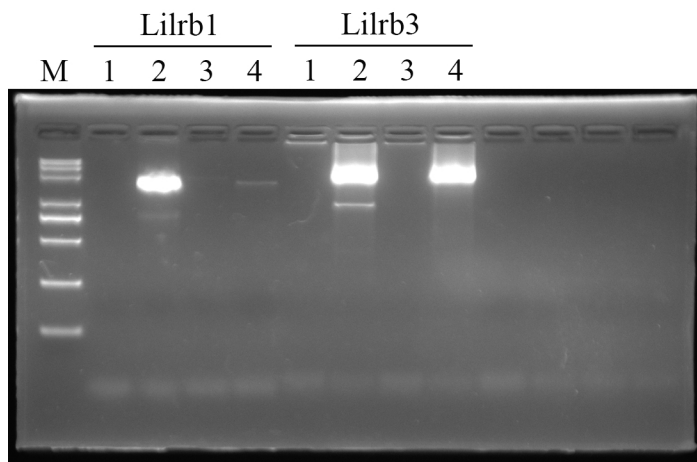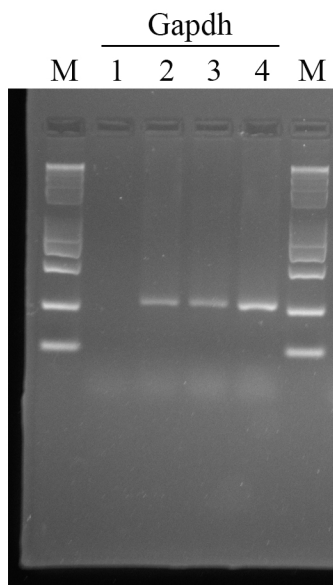

M: marker

1: NC (without cDNA templates)

2: Spleen

3: Bone marrow

4: Liver

Supplement: Supplementary file 6 — Supplementary Material 6 [file 12864_2023_9781_MOESM6_ESM.pdf]
